# Functional imaging derived ADHD biotypes based on deep clustering: a study on personalized medication therapy guidance

**DOI:** 10.1016/j.eclinm.2024.102876

**Published:** 2024-10-10

**Authors:** Aichen Feng, Dongmei Zhi, Yuan Feng, Rongtao Jiang, Zening Fu, Ming Xu, Min Zhao, Shan Yu, Michael Stevens, Li Sun, Vince Calhoun, Jing Sui

**Affiliations:** a Brainnetome Center and National Laboratory of Pattern Recognition, Institute of Automation, Chinese Academy of Sciences, Beijing, 100190, China; b School of Artificial Intelligence, University of Chinese Academy of Sciences, Beijing, 100049, China; c State Key Laboratory of Cognitive Neuroscience and Learning, Beijing Normal University, Beijing, 100875, China; d Peking University Sixth Hospital/Institute of Mental Health, National Clinical Research Center for Mental Disorders (Peking University Sixth Hospital), Beijing, 100191, China; e Department of Radiology and Biomedical Imaging, Yale University, New Haven, CT, USA; f Tri-institutional Center for Translational Research in Neuroimaging and Data Science (TReNDS), Georgia Institute of Technology, Emory University and Georgia State University, Atlanta, GA, 30303, United States; g Department of Psychiatry, Olin Neuropsychiatry Research Center, Institute of Living, Hartford Healthcare Corporation, Hartford, CT, United States; h Department of Psychiatry, Yale University School of Medicine, New Haven, CT, United States

**Keywords:** Attention deficit hyperactivity disorder (ADHD), Graph convolutional network (GCN), Deep clustering, Biological subtype detection, Adolescent brain and cognitive development (ABCD) study

## Abstract

**Background:**

Attention deficit hyperactivity disorder (ADHD) is one prevalent neurodevelopmental disorder with childhood onset, however, there is no clear correspondence established between clinical ADHD subtypes and primary medications. Identifying objective and reliable neuroimaging markers for categorizing ADHD biotypes may lead to more individualized, biotype-guided treatment.

**Methods:**

Here we proposed a graph convolution network for biological subtype detection (GCN-BSD) using both functional network connectivity (FNC) and non-imaging phenotypic data for ADHD biotype. We applied GCN-BSD to ADHD patients from the ABCD study as the discovery dataset and a validation ADHD dataset with longitudinal medication treatment from Peking University Sixth Hospital.

**Findings:**

We identified two biotypes based on 1069 ADHD patients selected from Adolescent Brain and Cognitive Development (ABCD) study, which were validated on independent ADHD adolescents undergoing longitudinal medication treatment (n = 130). Interestingly, in addition to differences in cognitive performance and hyperactivity/impulsivity symptoms, biotype 1 demonstrated a significantly better recovery rate in psychosomatic problems score (p < 0.05, baseline symptom score adjusted) when treated with methylphenidate than with atomoxetine.

**Interpretation:**

Our results suggested that such an imaging-driven, biotype-guided approaches hold promise for facilitating personalized treatment of ADHD and exploring possible boundaries through innovative deep learning algorithms to improve medication treatment effectiveness.

**Funding:**

Science and Technology Innovation 2030 Major Projects, the 10.13039/501100001809National Natural Science Foundation of China, the Startup Funds for Talents at 10.13039/501100002726Beijing Normal University, 10.13039/501100002858China Postdoctoral Science Foundation, and the 10.13039/100000002National Institutes of Health.

**Research in context**

**Evidence before this study**

We conducted a literature search in PubMed, bioRxiv, MedRxiv, ArXiv, IEEE Xplore, ScienceDirect for attention deficit hyperactivity disorder (ADHD) subtypes related analyses before May, 2024. Most studies: (1) focused on prior clinical subtype divisions based on core specific symptoms (ICD-9/10; DSM-4/5), i.e., Hyperactivity-Impulsivity (ADHD-HI), Inattention (ADHD-I) and the Combined (ADHD-C) subtype, and highlighted group-level differences via univariate statistic methods, (2) encompass investigations into multimodal neuroimaging and demographic features rather than symptoms, which can offer a novel perspective for unraveling the ADHD complexity and pathology brain function, however, the replicability and utility of the identified ADHD biotypes require further research.

**Added value of this study**

We proposed a novel framework, graph convolution network for biological subtype detection (GCN-BSD), that can jointly characterize brain imaging data and phenotypic association and further use this knowledge to guide disease biotype detection. Here, we identified two ADHD biotypes exhibit significant group differences in functional networks and multiple cognitive abilities and symptoms, especially in fluid intelligence and hyperactive/impulsive. We considered biotype 1 as a typical but mild ADHD pattern and biotype 2 as an atypical but more severe one. Additionally, we found a significant time–biotype interaction in multiple symptoms after medication, and biotype 1 demonstrated a significantly better recovery rate in psychosomatic problems score when treated with methylphenidate than with atomoxetine.

**Implications of all the available evidence**

This study indicates the validation of the frontoparietal circuits to serve as a key neuroimaging signature to ADHD and provide the first evidence for the connection from the cerebellum to the fusiform gyrus to be used as a biomarker in the uncommon biotype, helps move forward from a conventional biotype detection approach to the use of a more flexible deep learning-based analysis. To summarize, this study develops a more flexible deep learning-based biotype identification framework that aims to provide support for personalized efficacy treatment of adolescent ADHD.

## Introduction

Disease heterogeneity has been a critical challenge for precision diagnosis and treatment, especially in neuropsychiatric diseases. As a highly complex and heterogeneous disorder with childhood onset,^1^ attention deficit hyperactivity disorder (ADHD) is usually divided into three subtypes based on core specific symptoms, i.e., Hyperactivity-Impulsivity (ADHD-HI), Inattention (ADHD-I) and the Combined (ADHD-C) subtype.^2^ Meanwhile, stimulant medications (e.g., methylphenidate (MPH), amphetamine) and non-stimulant medications (e.g., atomoxetine (ATX)) are two main categories of medications approved for treating ADHD. However, currently there is no clear medication treatment plan corresponding to each subtype, which is decided mainly relying on clinical experience and not always being effective.^3^^,^^4^ Reports have demonstrated that MPH can equally improve inattention symptoms in both ADHD-C and ADHD-I subtypes, while predominantly reducing hyperactivity in the former.^5^ Therefore, for ADHD patients with pure inattention, the optimal medication selection is challenging in clinical practice. Thus, the identification of imaging-based ADHD biological subtypes (biotypes) that may exhibit greater sensitivity to specific drug therapies holds promise for guiding a more personalized treatment approach with enhanced effectiveness.

Meanwhile, multiple functional magnetic resonance imaging (fMRI) analyses have been performed to study ADHD subtypes,^6^, ^7^, ^8^, ^9^, ^10^ though in a relatively highly piecemeal way, which most relied heavily on prior clinical subtype divisions based on pure symptoms^7^ and highlighted group-level differences via univariate statistic methods.^8^^,^^9^ For example, children with ADHD-C were found to have hyper-connectivity in the pre-default mode network and lower network modularity compared to health controls,^11^ whereas no such differences were found in the ADHD-I subtype. While between ADHD patient subtypes such as ADHD-C/HI and ADHD-I, the level of comorbidity, treatment response, and possible etiological factors were often compared.

Motivated by this, we aim to develop innovative MRI-compatible, deep learning approaches to identify reliable ADHD biotypes in addressing the afore-mentioned medical issues. Deep learning (DL) has gained wide attention in mental disorder analysis due to its capability in automatically learning useful feature expression from high dimensional neuroimaging data, though its application in ADHD is still rare. In biotype division, compared to traditional clustering methods, deep clustering is able to learn nonlinear properties, represent low-dimensional embeddings from high-dimensional neuroimaging input, and constrains these learned features to adapt to the clustering target, which maximizes the between-class distance and minimizes the within-class distance.^12^^,^^13^ Specifically, both convolutional neural network and generative adversarial network have been employed to identify biotypes, e.g., from mild cognitive impairment. However, one issue is that clustering methods often ignored the topological information of population networks,^13^^,^^14^ i.e., complex associations between individuals were not fully explored, which may lead to sub-optimal performance in biotype identification. Therefore, we are motivated to integrate deep learning advances such as GCN and deep K-means to identify reliable, multi-factorial signatures for imaging-derived ADHD biotypes; so we can take an initial step towards developing biotype-guided treatment recommendations. Furthermore, public imaging data of children offers unprecedented opportunities for brain disorder research. With the aid of publicly shared data from Adolescent Brain Cognitive Development (ABCD)^11^ study and local longitudinal medication treatment data, we have an excellent platform with sufficient statistical power and enriched multi-factors (fMRI, demographics, cognition, symptoms) for testing and validating the proposed method and the identified ADHD biotypes.

**Fig. 1:**
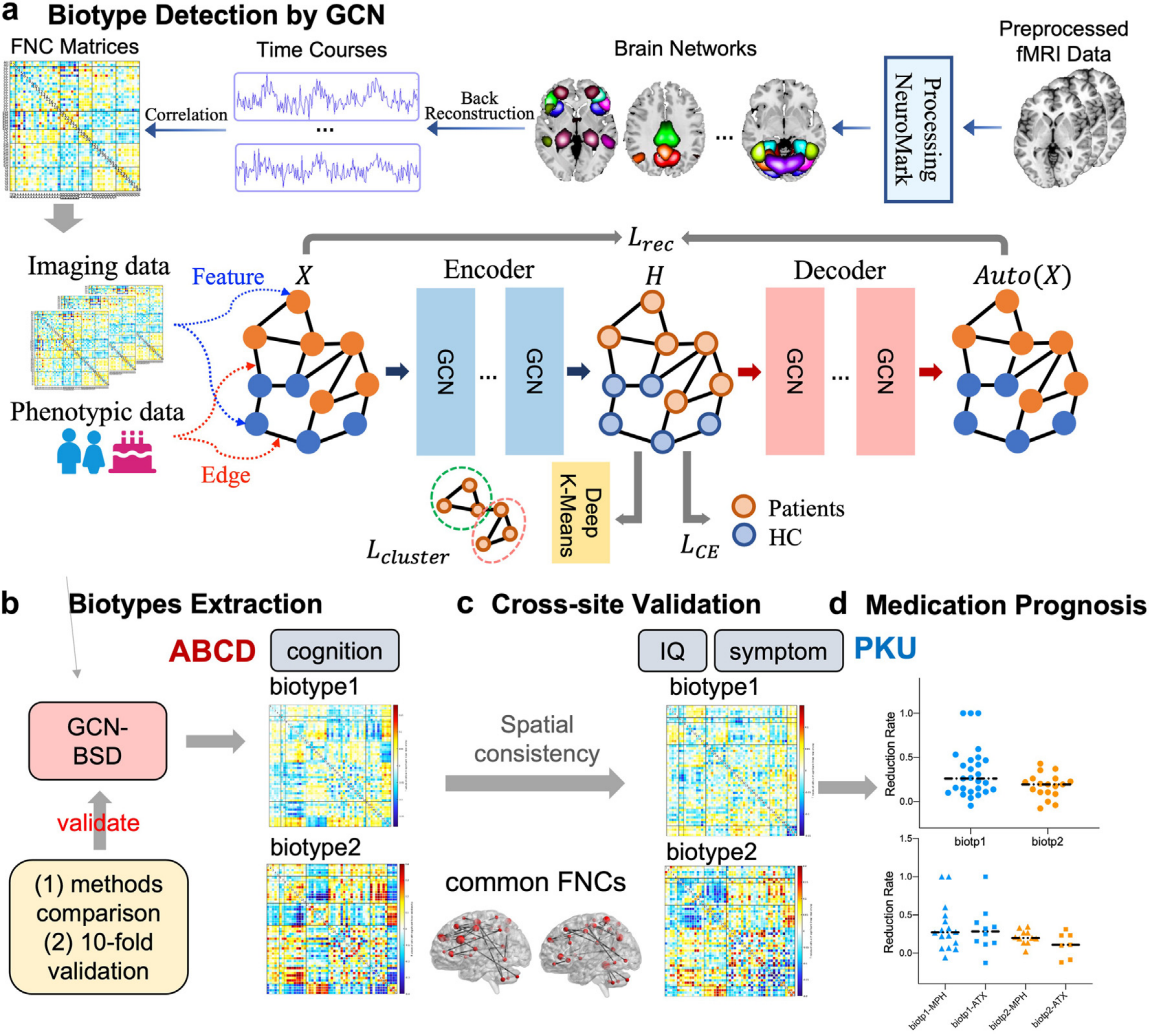
**Research outline**. (a) The framework of GCN-BCD, including functional imaging feature extraction——functional network connectivity (FNC) derivation and the model of GCN + deep k-means using population graph constructed via FNC and phenotypic data. (b) Biotype detection based on GCN-BSD applied in the ABCD dataset resulting in 2 biotypes. Evaluate the results by clustering method comparison and 10-fold cross-validation. (c) Validate the identified biotypes in PKU dataset through spatial consistency and extract the FNC biomarker. (d) Comparison of the symptom relief and treatment outcome of two biotypes either treated by MPH or ATX using longitudinal data from PKU. Collectively, this is a novel attempt to identify and validate ADHD biotypes that might push the boundaries of what is possible through innovative deep learning algorithms and assist in clinical medication selection to enhanced cure rates.

Consequently, we proposed a graph convolution network for biological subtype detection (GCN-BSD) using both functional imaging and non-imaging phenotypic data. As illustrated in Fig. 1, we first construct a population graph based on functional network connectivity (FNC, Fig. 1a) and phenotypic information (age, gender) to build individual mappings (see detailed FNC and network derivation in Method section). We applied GCN-BSD to learn clustering embeddings that are both group-discriminative between ADHD and controls, as well as adapted to the clustering constraint through K-Means loss. We selected 1069 ADHD patients from 11,875 children aged 9–11 from the ABCD study as the discovery dataset and a validation ADHD dataset with longitudinal medication treatment from Peking University Sixth Hospital (PKU6, 130 ADHD, 105 controls, aged 9–15), both with fMRI, cognitive metrics, and symptom records. Collectively, this is a novel attempt to identify and validate ADHD biotypes that might push the boundaries of what is possible through innovative deep learning algorithms and assist in clinical medication selection to enhanced cure rates.

## Methods

### Study design

A population graph was first constructed based on functional network connectivity and phenotypic information (age, gender) to build individual mappings; where FNCs serve as the feature of the nodes and the similarities between two subjects, which were abstracted from gender and age serve as edges. Then we applied GCN-BSD to learn embeddings that are both group-discriminative between ADHD and controls, as well as adapted to the clustering constraint through K-Means loss (Fig. 1a). We selected 1069 ADHD patients from the ABCD study as the discovery dataset, identified K = 2 for biotype division via the cluster sum of square (CSS) using the elbow method. We evaluated the clustering performance of 4 algorithms with GCN-BSD based on Davies-Bouldin Index (DBI) and Calinski-Harabasz Index (CHI) and applied 10 realizations of 10-fold cross-validation to test the stability of our results (Fig. 1b). As a result, two ADHD biotypes were identified, manifesting with different FNC patterns and distinguishing cognitive abilities. Then we used 130 ADHD and 105 controls collected from Peking University Sixth Hospital as validation dataset to test the generalizability and potential clinical use of the identified biotypes. We tested the similarity in the FNC patterns of ADHD biotypes identified in ABCD and PKU. The most contributing FNCs and clinical records were compared dedicatedly between two biotypes (Fig. 1c). Specifically, biotype 1 presented milder symptoms while biotype 2 manifested more severe hyperactivity/impulsivity symptoms and worse cognitive levels. Finally, we compared the symptom relief and treatment outcome of two biotypes from 44 out of 130 ADHD patients either treated by MPH or ATX at PKU6 according to our division (Fig. 1d).

### The discovery ABCD cohort

The ABCD study (https://nda.nih.gov/abcd/) is an observation assessment of brain development in children in the US at 9–11 years of age to characterize psychological and neurobiological development from early adolescence to early adulthood.^11^ Our data were analyzed from the ABCD Study curated annual release 3.0, which contains baseline data from 11,875 children. We determined ADHD same as previous studies^15^ by past and present mental disorder diagnoses using parent-reported responses to the self-administered computerized Schedule for Affective Disorders and Schizophrenia for School-Age Children for DSM-5 (K-SADS-5).

### Independent PKU cohort

Another in-house dataset was included in this study as a validation. This children's cohort was approved by the Ethics Committee of Peking University Sixth Hospital. A total of 130 children with ADHD and 105 HCs (6–15 years old) were recruited. 44 patients received medical treatment who were unmedicated before, 17 of them were treated with atomoxetine (ATX), and 27 of them were treated with methylphenidate (MPH), and we recorded their symptom scales at baseline, week 1, week 2, week 3, week 4, and week 8. The diagnosis was made by a senior psychiatrist based on the Schedule for Affective Disorders and Schizophrenia for School-Age Children-Present and Lifetime version (K-SADS-PL), which is a clinical and semi-structured interview based on the Diagnostic and Statistical Manual of Mental Disorders-Fourth Edition (DSM-IV). The severity of inattentive symptoms, hyperactive/impulsive symptoms, and total ADHD symptoms of all subjects were evaluated by the ADHD Rating Scale-IV (ADHD RS-IV). Conners' Parent Rating Scale (CPRS) was used to assess the hyperactive/impulsive symptoms (see more details in the Supplementary file). The demographic information was listed in Table S2.

### Image acquisition and processing

The resting-state fMRI data from two datasets were preprocessed with a combination of FMRIB Software Library v6.0 toolbox and Statistical Parametric Mapping 12 toolbox, including rigid body motion and distortion correction, spatial normalization into Montreal Neurological Institute space, and smoothing with a 6-mm full width at half maximum Gaussian kernel (please see more details in Supplementary file). We decomposed the preprocessed MRI by a fully automated spatially constrained ICA run on each individual subject separately based on the neuromark_fMRI_1.0 template (available in GIFT; http://trendscenter.org/software/gift and as a separate download at http://trendscenter.org/data) and pipeline used in.^16^^,^^17^ For this template, fifty-three independent component networks (ICNs) were identified and arranged into 7 functional networks, including the subcortical (SC) (5 components), auditory (AU) (2 components), sensorimotor (SM) (9 components), visual (VI) (9 components), cognitive control (CC) (17 components), default mode (DM) (7 components), and cerebellar (CB) (4 components) networks. FNC is calculated using Pearson correlation, resulting in a 53 × 53 matrix for each subject. We extracted the upper triangle elements of the matrix as FNC features, namely, each subject has an FNC vector in the dimension of (53 × 52)/2 = 1378. Participants from the ABCD dataset were selected based on the availability of non-imaging measures and high-quality neuroimaging data. The quality assessment of the neuroimaging data was based on the “Quality Control and Recommended Inclusion Criteria for Structural Data” provided as a document in the “Release Notes Imaging Instruments” folder. Participants were excluded from the present analyses because of incomplete rsMRI data at baseline and/or because brain scans did not pass the ABCD Study's quality control. After all quality control, we have 1069 ADHD participants included (see more details in the Supplementary file). And 1164 demographically matched HCs were included in our study. Their demographic and cognitive information were listed in Table S1.

### Cognitive abilities and symptom scales

The ABCD baseline cognitive battery assessed by a well-validated National Institute of Health Toolbox was used as evaluation in this study,^18^ which covers Picture Vocabulary Test Score (PVT); Flanker Inhibitory Control and Attention Test Score (FICA); List Sorting Working Memory Score (LSWM); Dimensional Change Card Sort Test Score (DCCS); Pattern Comparison Processing Speed Test Score (PCPS); Picture Sequence Memory Test Score (PSM); Oral Reading Recognition Test Score (ORR); Cognition Fluid Composite Score; Crystallized Composite Score; Cognition Total Composite Score. For environmental status, we considered caregiver education and family incomes from ABCD Parent Demographics Survey. Age-corrected T-scores were used in this study where higher values correspond to better performance.

For patients from PKU, the severity of inattentive symptoms, hyperactive/impulsive symptoms, and total ADHD symptoms of all subjects were evaluated by the ADHD Rating Scale-IV (ADHD RS-IV). Besides, the Conners’ Parent Rating Scale (CPRS) was used to assess the symptoms in children participants with ADHD. The CPRS is a widely used instrument for screening and evaluating ADHD-related symptoms as well as other behavioral problems frequently associated with ADHD in children. It contains 48 items and can be divided into six factors: conduct problems, learning problems, psychosomatic problems, impulsivity–hyperactivity, anxiety, and ADHD index. Verbal IQ, performance IQ, and full-scale IQ were measured by the Wechsler Child/Adult Intelligence Scale, Third Edition.

### GCN for biotype detection

GCN-BSD is an autoencoder-based GCN model. The proposed architecture consists of three main elements: an encoder, an embedding layer, and a decoder, as shown in Fig. 1b. The encoder and decoder are connected via the embeddings. By putting the embeddings into the fully connected layer, we adopted the binary cross entropy function to learn group-discriminative features, which identified the subject labels as patients or healthy controls for all subjects. Besides, we put the patient subgraph with the connection unchanged into the clustering layer for biotype detection. The deep K-Means loss function is adopted as the clustering loss. To better extract high-resolution embeddings, we also adopted the reconstruction loss. The GCN-BSD can then be formulated as minimizing the following function:

$$L=\sum \gamma_{1}L_{rec}\left(X\right)+\gamma_{2}L_{CE}\left(X\right)+\gamma_{3}L_{cluster}\left(X\right)$$

where γ_1,_ γ_2_ and γ_3_ regulate the trade-off among seeking good representation for X, which is set for biotype clustering, meanwhile is faithful to the original feature and maintains label information. GCN-BSD was implemented with Pytorch and optimized using Adam optimizer. Hyperparameters were set as: γ_1_ = 0.01, γ_2_ = 0.01, γ_3_ = 0.1, and learning rate was 0.001. More details can be found in the Supplementary file. Firstly, GCN-BSD was adopted in the ABCD dataset to identify ADHD biotypes. The number of clusters K was decided by the cluster sum of square (CSS) using the elbow method.

### Clusters/biotypes validation

We tested the reliability of two clusters/biotypes within ABCD data by performing 10 times of 10-fold cross-validation. We also tested the contribution of the population graph and imaging data itself by using random data as the nodes’ feature instead of using FNC. More details can be found in the Supplementary file.

To evaluate the clustering performance, we compared four alternative clustering methods with GCN-BSD, including (1) agglomerative clustering, (2) conventional K-Means, (3) DNN with deep K-Means, and (4) autoencoder GCN with K-Means. The clustering performance was measured by two internal cluster evaluation measures: DBI and CHI, and one performance indicator: ABCD cognitive battery. Statistical analyses were performed among ADHD biotypes and HC. A two-sample t-test was performed for all measures between biotypes and HC with age and gender as the nuisance covariates. The p-values were corrected for multiple comparisons using FDR correction.

To further evaluate the stability of identified ABCD biotypes, we also used a more restricted definition from Cordova's^6^ and consider medication status and family relatedness. More details can be found in the Supplementary file.

### Cross-site validation

To validate the constancy of the identified ADHD biotype patterns, we used the mean FNC feature of two biotypes as templates and projected them into an independent dataset from PKU to extract similar biotype patterns. Specifically, for each subject in PKU, we calculated the Euclidean distance between individual FNC and two FNC templates, and then we divided the subject into a subgroup where the template showed more similarity than the other. The two-sample t-test, and effect size Cohen's d were evaluated to calculate the group differences between two biotypes of the symptom scales. We used two sample t-test with age and gender as the nuisance covariates. Note that sites and scanners were regressed out before statistical analysis. The mean FD was added as a nuisance covariate in the FNC analysis.

The top 100 discriminative FNC in two biotypes were identified by two-sample t-tests in ABCD and PKU, respectively. We grouped the 100 FNCs into 7 brain networks according to the NeuroMark pipeline to analyze the functional connectivity on a large scale. Moreover, to visualize the consistent brain patterns, we picked the top 100 FNCs which overlap between two separate datasets and obtained brain functional patterns for two biotypes. We further calculated the correlations between the identified overlapped FNC and multiple scales in two datasets, separately, in order to study the association between FNC and multiple symptoms.

### Different medication sensitivity between two biotypes

In order to explore whether the identified biotypes respond differently to medical treatment, linear mixed-effects modeling^19^ was performed using the lme4 package in R^20^ to compared the treatment outcome under medication treatment of ATX and MPH after weeks. 44 out of 130 ADHD patients from PKU were recorded with longitudinal symptom scores, who were divided into two biotypes according to the previous results. We calculated the reduction rate for RS-IV and CPRS from week 1 to week 8, with baseline scales being regressed out. To incorporate a week-by-biotype interaction term, the LME model included fixed effects for time, biotypes, and their interaction; children's reduction rate as the dependent variable; and a subject-specific random intercept and slope. Furthermore, to test whether there are differences in the benefits of the drug between 2 biotypes, we used the LME model as: score ∼ week × sub + med × sub + week × med + (1|ID), where (1|ID) means a subject ID-specific random intercept and slope. Statistical significance for main effects and interactions was assessed using the ANOVA F-test and set at a p-values of 0.05. The raw reduction rate and regressed reduction rate are provided in the Supplementary file.

### Ethics

This study was approved by the Ethics Committee of Peking University Sixth Hospital, and the scientific review form, research protocol (approval number SF20221205), informed consent form for children and parents (approval number SF20221205) were reviewed and approved at the meeting on December 26, 2022. The ethical batch number is: 2022 No. 78 (2022-11-16-4). This study was conducted following the ethical principles of the Declaration of Helsinki and all applicable local regulations. All patients provided written informed consent prior to enrolment.

### Role of the funding source

The funding source had no role in the design, execution, analyses, interpretation of the data, or decision to submit results.

## Results

### The validation of GCN-BSD

**Fig. 2:**
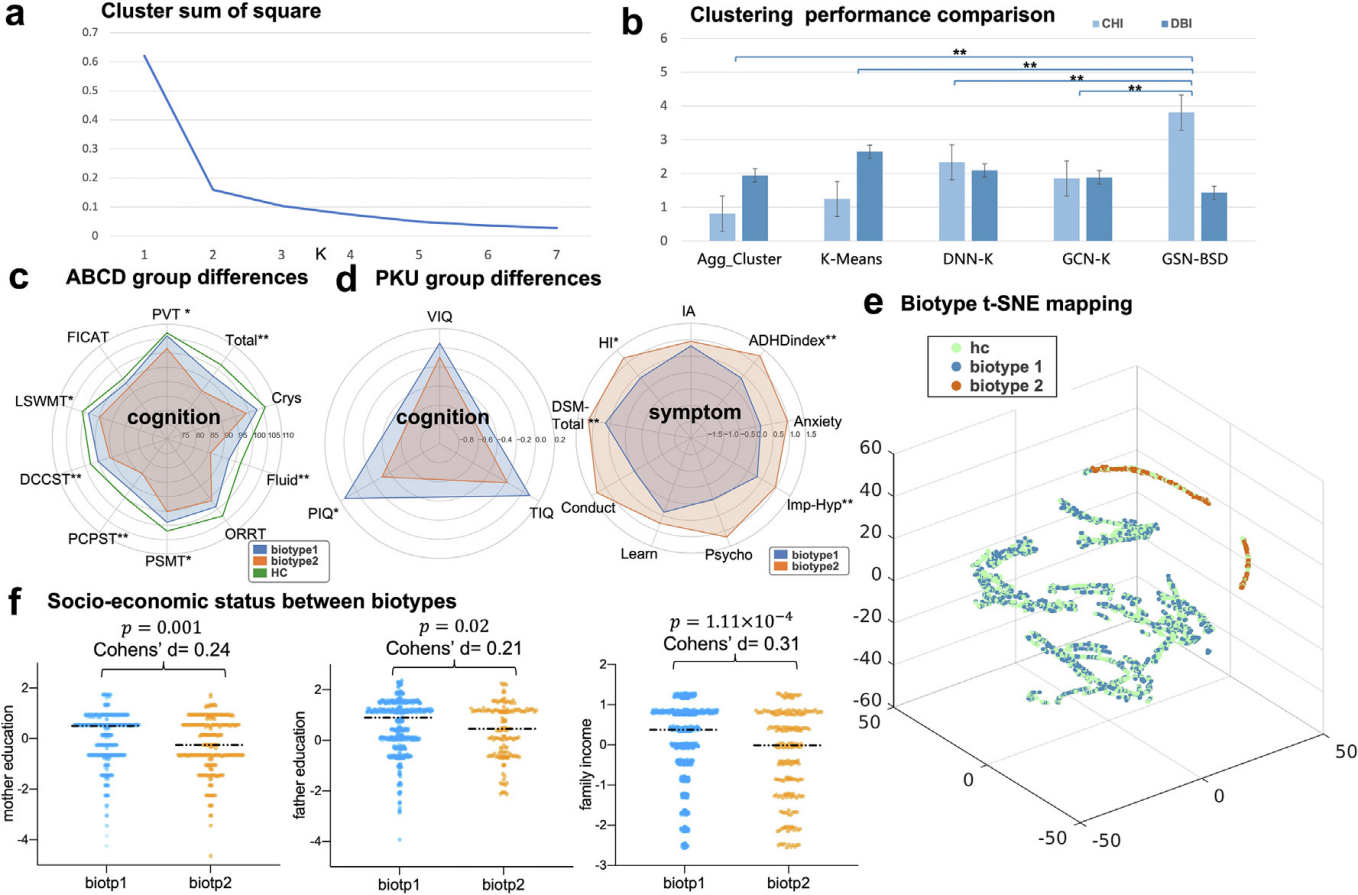
**ADHD biotypes detection results**. **(a)** Cluster sum of square (CSS) across the number of clusters. **(b)** GCN-BSD shows the best clustering performance among the 5 methods, i.e., highest CHI and lowest DBI. **(c)** Mean cognitive scores for biotype 1 (blue), biotype 2 (yellow), and HC (green) in ABCD dataset, note that a higher score means better cognitive abilities. **(d)** Mean cognitive scores for biotype 1 (blue) and biotype 2 (yellow) in the PKU dataset, note that a higher score means better cognitive abilities in the left subgraph and a higher score means severer symptoms in the right subgraph. **(e)** Clustering results of the embedding features learned from GCN-BSD for ADHD biotypes and HC visualized by t-SNE, where the embedding features of biotype 1 and biotype 2 are separatable. (**f**) Significant group differences between biotype 1 and biotype 2 considering age-corrected environmental exposures including mother education, father education and family income.

Two ADHD biotypes were identified, and the number of clusters was decided by the SSE using the elbow method, as shown in Fig. 2a. Through plotting the SSE as a function of the number of clusters k and picking the elbow of the curve, we chose k = 2 as the optimal number of clusters/subtypes. As a result, 821 children were identified as biotype 1, and the rest of them were identified as biotype 2.

The performance of our GCN-BSD for significant characteristics along with conventional methods and deep methods with the input of FNC was reported in Table 1 and Fig. 2b. The clustering performance is evaluated concerning two standard measures: DBI and CHI. As shown in Fig. 2b, GCN-BSD reached the lowest DBI and highest CHI. Meanwhile, GCN-BSD achieved the smallest p-values in several cognitive characteristics. Notice that the results of the compared methods only showed the similar trends as GCN-BSD, the number of each cluster may not be the same. In general, compared with the traditional clustering methods and naïve deep learning methods, GCN-BSD achieves better performance by its abstracted features and GCN module.

**Table 1:** Comparison of p-value in significant cognitive characteristics between 2 biotypes.

Cognitive measures	p-values of 2 sample t-test between biotype 1 and biotype 2				
	Agg-Cluster	K-Means	DNN-K means	GCN-K means	GCN-BSD
PVT	0.07	0.02	2.83 × 10^−3^	0.01	**1.22** × **10**^**−**^**^4^**
LSWMT	0.03	0.01	8.83 × 10^−3^	0.02	**1.35** × **10**^**−**^**^4^**
DCCST	2.71 × 10^−3^	1.67 × 10^−4^	3.01 × 10^−4^	1.21 × 10^−4^	**2.87** × **10**^**−**^**^5^**
PCPST	0.02	4.36 × 10^−5^	2.27 × 10^−5^	2.38 × 10^−4^	**1.89** × **10**^**−**^**^6^**
PSMT	0.10	0.01	0.03	0.04	**8.57** × **10**^**−**^**^4^**
Fluid cognition	4.66 × 10^−4^	1.29 × 10^−6^	7.25 × 10^−6^	1.86 × 10^−7^	**3.09** × **10**^**−**^**^8^**
Total cognition	8.92 × 10^−4^	1.12 × 10^−5^	1.25 × 10^−5^	3.49 × 10^−6^	**2.61** × **10**^**−**^**^7^**

Note: PVT, picture vocabulary test; LSWMT, list sorting working memory test; DCCST, dimensional change card sort test; PCPST, pattern comparison processing speed test; PSMT, picture sequence memory test. GCN-BSD achieved the most significnat p-value.

### ADHD biotypes extracted from ABCD

**Fig. 3:**
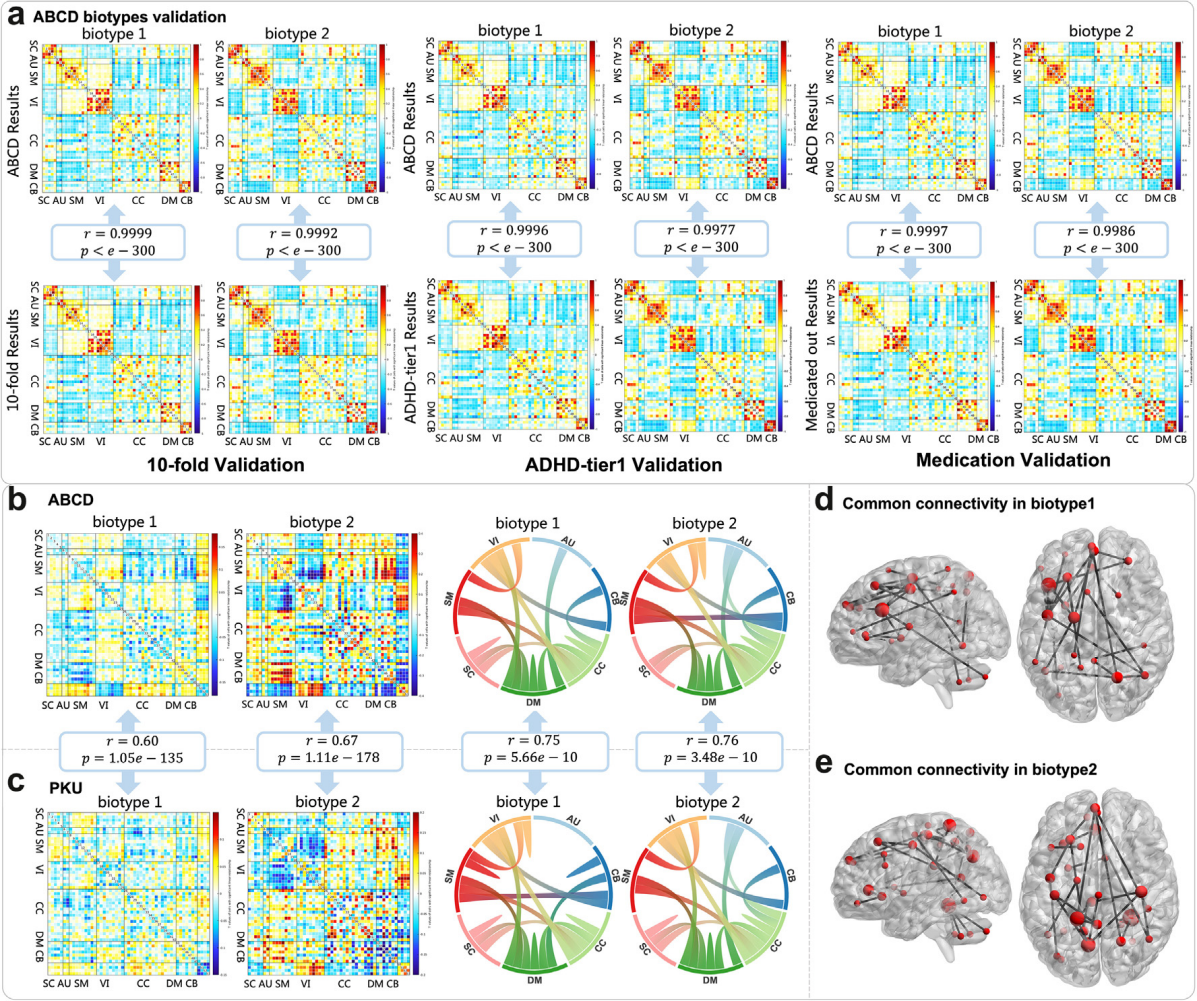
**ADHD biotypes detection results**. **(a)** The validations of ABCD biotypes considering 10-fold cross-validation, additional ADHD and medication restrictions show high correlation with the original biotypes (r = 0.99, p < 10^−300^). **(b)** The mean differences of FNC between 2 biotypes and HC in ABCD and Top 100 connectivity patterns of biotype 1 and biotype 2 in the view of the contribution of functional networks. **(c)** The mean FNC differences between 2 biotypes-HC in PKU show high correlation with results from ABCD (r > 0.6, p < 10^−100^ for both biotypes). Top 100 connectivity patterns of biotype 1 and biotype 2 in the view of the contribution of functional networks in PKU, showing high correlation with results from ABCD (r = 0.75 for biotype 1, r = 0.76 for biotype 2, p < 6 × 10^−10^). **(d)** Overlapped top 100 discriminative FNCs for biotype 1 between ABCD and PKU. **(e)** Overlapped top 100 discriminative FNCs for biotype 2 between ABCD and PKU. FDR corrected.

Biotype 1 and biotype 2 have different connectivity patterns from HC. The mean differences of FNC between 2 biotypes and HC are shown in Fig. 3b. Fifty-three maximally independent component networks were arranged into 7 functional domains. Biotype 1 shows positive connectivity within DM and between SM, CC, and CB, between VI and SM, SC, and negative connectivity between SM and DM, VI and CB. Biotype 2 shows more severe abnormality among all networks, compared to biotype 1. It shows positive connectivity between SM and DM, VI and CB, and negative connectivity between VI, CB, and SM, between DM and CB.

The two biotypes and demographically matched healthy controls were compared in cognitive measures (Table 2). Overall, across all ten cognitive scores, HC has the highest mean values, and biotype 1 has the lowest mean values among the three groups (Fig. 2c). The two biotypes are significantly different in seven out of 10 cognitive measures. Notably, fluid cognition p = 3.09 × 10^−8^) and total cognition (p = 2.61 × 10^−7^) showed the most significant difference between the two biotypes. Besides, our results suggested that significant group differences between biotype1 and biotype 2 were identified considering both mother and father education and family income (Fig. 2f: mother education: p = 0.001, father's education: p = 0.02, family income: p = 1.11 × 10^−4^, all FDR corrected).

**Table 2:** Demographic and cognitive characteristics of the biotypes from ABCD.

Characteristics	biotype 1	biotype 2	p-value	Cohen's d
Num	821	248		
Age (months)	118.88 ± 7.61	118.37 ± 7.27	0.33	
Gender (M/F)	523/289	174/74	0.11	
PVT	105.97 ± 15.81	101.62 ± 15.37	1.22 × 10^−4^	0.28
LSWMT	98.74 ± 14.30	94.70 ± 14.52	1.35 × 10^−4^	0.28
DCCST	95.21 ± 14.77	90.95 ± 13.68	2.87 × 10^−5^	0.30
PCPST	91.62 ± 22.42	84.66 ± 22.14	1.89 × 10^−6^	0.31
PSMT	98.94 ± 15.43	95.20 ± 15.36	8.57 × 10^−4^	0.24
Fluid Cognition	92.72 ± 17.00	85.98 ± 16.32	3.09 × 10^−8^	0.40
Total Cognition	97.18 ± 17.20	90.72 ± 16.95	2.61 × 10^−7^	0.38

Note: PVT, picture vocabulary test; LSWMT, list sorting working memory test; DCCST, dimensional change card sort test; PCPST, pattern comparison processing speed test; PSMT, picture sequence memory test. FDR corrected.

The 10-fold cross-validation showed that the mean FNC patterns existed extremely high correlation between ABCD dataset and 10-fold subsets (Fig. 3a, r = 0.99, p < 10^−300^). When we implemented additional restrictions based on the screening criteria referenced in the Cordova's previous work^6^ or excluded the medicated participants, the original biotype FNC patterns also showed high correlation with the ADHD-tier 1 and medicated out biotype FNC patterns (Fig. 3a, r = 0.99, p < 10^−300^). See more details in the Supplementary.

### ADHD biotypes validated in PKU cohort

We further tested whether the identified connectivity pattern was consistent in the independent data cohort. By projecting the identified ADHD biotypes from ABCD into the PKU dataset, subjects were divided into 2 subsets. We detected the top 100 discriminative connectivity from 1378 FNCs in two biotypes by using two-sample t-tests to estimate the group differences of FNC features. To explicitly characterize the contribution of each functional network, we grouped the 53 nodes into 7 networks according to the NeuroMark pipeline, as shown in Fig. 3b and c. Connectivity patterns of biotype 1 and biotype 2 were highly replicable from ABCD to PKU (correlation: r = 0.75, p = 5.66 × 10^−10^ for biotype 1, r = 0.76, p = 3.48 × 10^−10^ for biotype 2), and regions in the DM and CC exhibited the greatest involvement. For biotype 1, the results showed that the connectivity among CC, DM, SC, SM, and VI is overrepresented and repeatable. While for biotype 2, apart from the connectivity among multiple networks and VI, the overrepresented connections between AU and CC, DM, between CC and CB are also validated between datasets. Then we expanded these to the whole FNC, the mean differences between biotypes and HCs in PKU still reveal a clear correlation to ABCD, where r = 0.60, p = 1.05 × 10^−135^ for biotype 1 and r = 0.67, p = 1.11 × 10^−178^ for biotype 2, as shown in Fig. 3c.

In PKU, two-sample t-tests and effect size Cohen's d was used to estimate the group differences of the IQ scale and symptom scale, see Table 3. Performance IQ measured by the Wechsler Child/Adult Intelligence Scale distinguished itself (p = 0.01). And we noticed that hyperactive/impulsive symptoms and total ADHD symptoms from RS-IV, and impulsivity–hyperactivity factor and ADHD index from CPRS showed significant differences between biotype 1 and biotype 2 (p = 4.97 × 10^−4^ for RS-hyperactive/impulsive, p = 3.82 × 10^−3^ for RS-total, p = 8.67 × 10^−3^ for CPRS-impulsivity–hyperactivity, p = 0.01, for CPRS-ADHD index).

**Table 3:** Demographic and clinical characteristics of the biotypes from PKU.

Characteristics	biotype 1	biotype 2	p-value	Cohen's d
Subj num	89	41		
Age (years)	9.61 ± 2.88	9.41 ± 2.56	0.49	
Gender (M/F)	82/21	32/9	0.84	
RS_HI	18.64 ± 4.27	22.22 ± 5.16	4.97 × 10^−4^	0.68
RS_Total	45.14 ± 6.79	48.82 ± 6.42	3.82 × 10^−3^	0.56
PIQ	107.41 ± 15.28	100.89 ± 11.59	0.01	0.48
CPRS_IH	4.94 ± 2.27	6.33 ± 2.75	8.67 × 10^−3^	0.55
CPRS_ADHD-Index	12.96 ± 4.18	15.23 ± 4.66	0.01	0.51

Note: RS_HI, RS-hyperactive/impulsive symptoms; RS_Total, RS-total ADHD symptoms; PIQ, Performance IQ; CPRS_IH, CPRS-impulsivity–hyperactivity factor; FDR corrected.

### FNC pattern consistency across datasets

Moreover, as shown in Fig. 3d and e, we extracted the overlapping FNCs between ABCD and PKU, resulting in 26 FNCs for biotype 1 and 27 for biotype 2. Both in the biotype 1 and biotype 2, the connections between the superior parietal lobule and posterior cingulate cortex, precuneus in DM, between the precuneus and anterior cingulate cortex, superior frontal gyrus, between the precentral gyrus and superior frontal gyrus are overrepresented. Separately, the key nodes in biotype 1 are the paracentral lobule, superior parietal lobule, cerebellum, frontal gyrus, and precentral gyrus, while in biotype 2 those are fusiform gyrus, posterior cingulate cortex, precuneus, anterior cingulate cortex and cerebellum. For biotype 1, the connectivity within the frontal gyrus and their connections with other regions including the cerebellum, middle temporal gyrus, paracentral lobule, precentral gyrus, and inferior parietal lobule are overrepresented. While for biotype 2, FNCs exhibited considerably denser patterns and greater degrees in DM regions including the precuneus, anterior cingulate cortex, and posterior cingulate cortex. And connectivity between the cerebellum and fusiform gyrus, and between the insula and anterior cingulate cortex are overrepresented for biotype 2.

The consistent discriminative FNCs between biotype 1 and biotype 2 were highly correlated with several cognition scales. The correlation results of the connectivity between the middle temporal gyrus and paracentral lobule, between the superior parietal lobule and posterior cingulate cortex, and the specialized connectivity cerebellum-fusiform gyrus in biotype 2 are listed in Table S4.

### Comparison of medication effect of two biotypes

**Fig. 4:**
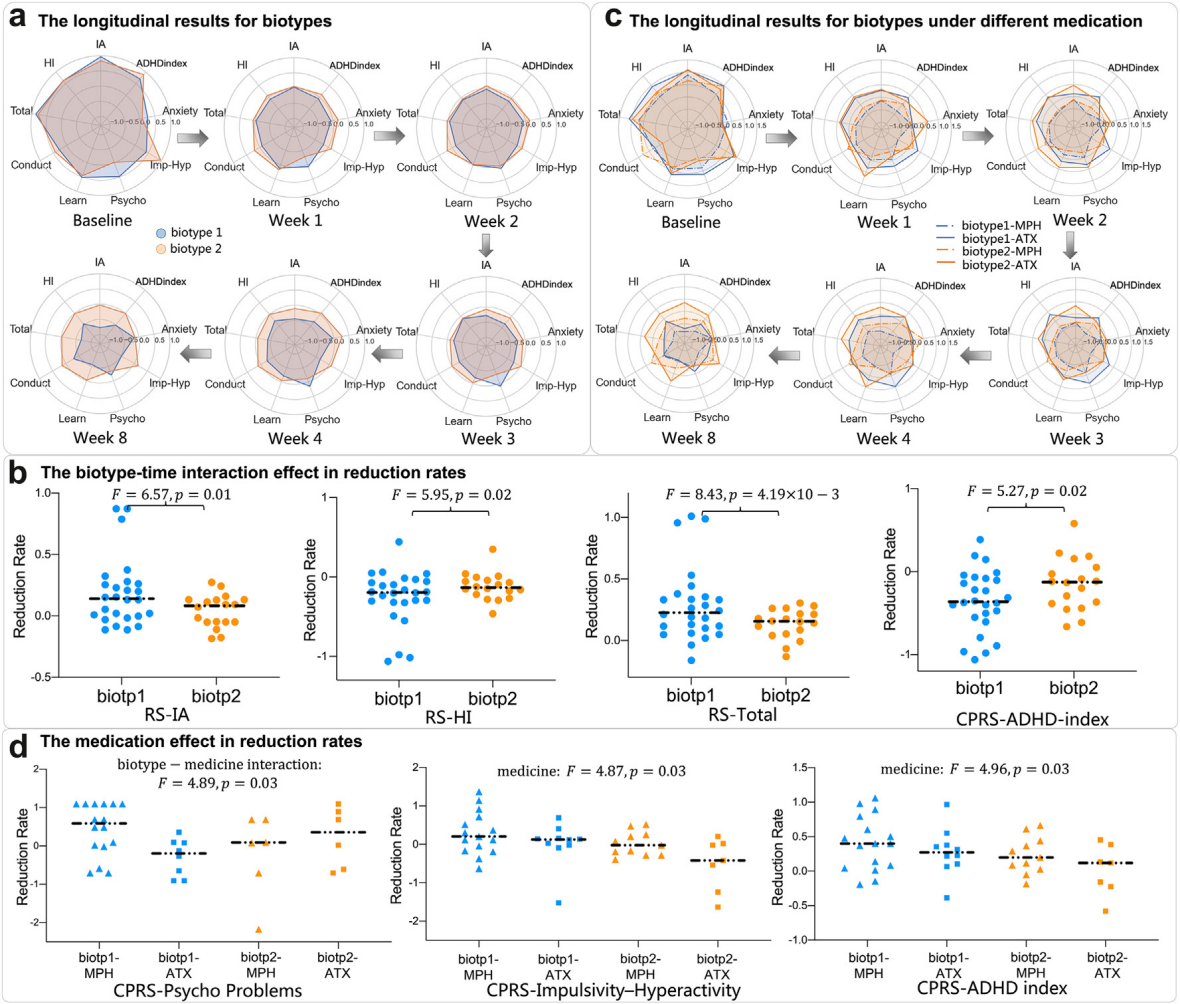
**The longitudinal symptom changes for ADHD biotypes under different medication**. Symptom changes of two identified ADHD biotypes during 1 week–8 weeks when being treated with either MPH or ATX. **(a)** The overall 9 domains of symptom change measured by RS-IV and CPRS from baseline to week 8, where blue line denotes biotype1 and orange line denotes biotype 2. Note that a lower score (smaller range) means milder symptoms. From the baseline to week 8, biotype 1 showed a steady decline in all ADHD symptom domains, while the symptom reduction for biotype 2 stagnated after week 4. **(b)** Biotype 1 show significantly better recovery rate than biotype 2 on symptom domains including RS-IA (p = 0.01), RS-HI (p = 0.02), RS-total (p = 4.19 × 10^−3^), and CPRS-H-index (p = 0.02). **(c)** Nine domains of symptom change measured by RS-IV and CPRS for two ADHD biotypes treated either by MPH (dotted line) or ATX (solid line) from week 1 to week 8. **(d)** The significant biotype–medicine interaction effect in CPRS-psychosomatic problems (p = 0.03), and significant medicine effect in CPRS-IH (p = 0.03) and CPRS-H-index (p = 0.03).

One of the important applications of biotype detection is to observe their clinical progression and differences under treatment; therefore, we further evaluated symptom improvement within the ADHD patients under the treatment of ATX and MPH in week 1, 2, 3, 4, 8. For ADHD children under medication from PKU, 27 of 44 patients were classified as biotype 1 and the rest of them as biotype 2. To test whether there are differences in the benefits of the drug between 2 biotypes, we used linear mixed effect (LME) model to rigorously evaluate the symptom reduction of RS-IV and CPRS in all 9 domains. The demographic information and differences in symptom reduction between the biotype 1 and biotype 2 are shown in the Tables S8 and S10 in Supplementary file. After medication, biotype 1 and biotype 2 showed similar symptom relief from week 1 to week 4 as shown in Fig. 4a. After week 4, biotype 1 still showed a steady decline, while biotype 2 showed a minor reduction in multiple symptoms. As shown in Fig. 4b, we found significant time–biotype interaction in inattention and hyperactive/impulsive domains such as RS-IA (F = 6.57, p = 0.01), RS-HI (F = 5.95, p = 0.02), RS-Total (F = 8.43, p = 4.19 × 10^−3^) and CPRS-H-index (F = 5.27, p = 0.02). Please see more validation and testing details in the Supplementary.

Furthermore, we analyzed the prognosis of using different medication in two biotypes. 16 of 26 subjects in biotype 1 and 11 of 18 subjects in biotype 2 took MPH, and the rest of them took ATX. The demographic information and differences in symptom reduction between the ATX group and MPH group are shown in the Tables S3 and S5 in Supplementary file. As shown in Fig. 4c, by using LME to estimate the two-way interaction among biotypes, medicine, and time, we found the significant biotype–medicine interaction effect in CPRS-psychosomatic problems (F = 4.89, p = 0.03). And we also found significant medicine effects in hyperactive/impulsive domains such as CPRS-IH (F = 4.87, p = 0.03) and CPRS-H-index (F = 4.96, p = 0.03). See more details in the Supplementary.

## Discussion

In this study, we proposed a novel GCN-based deep clustering framework, compared it with multiple clustering methods, validated its utility in ADHD biotype detection, and identified two clinically discrepant biotype patterns that were tested in two independent cohorts and show great potential for personalized medication. Biotype 1 contained more patients, presented milder symptoms, and overrepresented several wildly recognized brain aberrations including frontal gyrus and cerebellum. In contrast, biotype 2 included fewer patients, presented more severe symptoms especially hyperactive/impulsive, and showed greater degrees in regions from DM to SM, as well as the connectivity between the cerebellum and fusiform gyrus. Based on this, we considered biotype 1 as a typical but mild ADHD pattern and biotype 2 as an atypical but more severe one. To the best of our knowledge, this is a novel attempt to introduce population association into the identification of disorder biotypes and identified two ADHD biotypes showing significant group differences.

Disease heterogeneity has attracted much interest, for it elucidates the implicit pathological mechanisms and makes it possible to offer individual treatment options to patients from different subgroups. For example, Varol et al. present an algorithm HYDRA based on SVM to simultaneously identify a class of pathological samples and separate them into coherent subgroups.^21^ Building on HYDRA, Wen et al. proposed MAGIC to capture multi-scale representations of disorder subgroups and exploited a double-cycle optimization method.^22^ In contrast to existing clustering approaches presented in,^21^^,^^22^ we considered the population association by introducing population graph GCN into deep clustering, for heterogeneity is a property that can only be expressed at the population level instead of the individual level. Population graph GCN has been proven effective in disease classification by a number of previous studies.^23^ Our GCN-BSD not only outperformed conventional clustering methods but also significantly outperformed the deep learning-based clustering model. When using the publicly accessible dataset ABCD, lowest DBI and highest CHI were achieved in ADHD biotype detection. Both as traditional methods, K-Means showed decreased p-values in characteristics compared to agglomerative clustering. As expected, we observed that the DNN with K-Means and GCN with K-Means performed better than the two conventional approaches.

In addition, 10-fold cross-validation within cohort and validation in external cohort further indicated the effectiveness of our identified biotypes. As shown in Fig. 3 and Fig. S3, the two identified ADHD biotypes are quite consistent across two independent cohorts (ABCD and PKU) and validated in random data subsets. Though the results obtained from sub-sets may not be exactly the same as when using all the data, the two biotypes show almost the same FC patterns. ADHD biotypes identified from ABCD not only make sense in the objective index and show significance in cognitive scales, but also be validated in PKU. Interestingly, besides consistent with the cognitive results in ABCD, biotypes derived from PKU were also differentiable in performance IQ and more symptoms including hyperactivity/impulsivity.

The pattern of biotype 1 is in accord with the classical ADHD model as a disorder of deficient frontoparietal and multiple regions activation, while biotype 2 expresses an unconventional pattern. Regarding the specific abnormalities identified in biotype 1, this typical but mild biotype is overrepresented in multiple connectivity from DM to SM and CC, besides, the anterior cingulate cortex, cerebellum, paracentral lobule, and frontal gyrus also present as the key nodes. The frontal gyrus is hypo-activated in various tasks ranging from working memory to time discrimination.^24^^,^^25^ ADHD has long been thought to reflect dysfunction of prefrontal–striatal–cerebellar circuitry, but accumulating evidence suggests that the prefrontal–striatal model of ADHD should be extended to include other circuits and their interrelationships from the perspective of systems neuroscience. Consistent with the previous studies,^26^ the biotype 1 supported a model that ADHD-related dysfunctions are not only involved in higher-level cognitive-behavioral functions, such as the frontoparietal and default networks, but also in sensorimotor processes, including SM. Note that all the remaining regions implicated in the prefrontal–striatal–cerebellar model of ADHD are components of the frontoparietal circuit known as the executive control circuit.^27^ As for the unconventional biotype 2, we identified connectivity which not overrepresented in biotype 1. We noticed that besides the frontoparietal circuit, the special connectivity cerebellum-fusiform gyrus is highly correlated with multiple measures, including total intelligence, fluid intelligence, crystallized intelligence, inattentive and hyperactive/impulsive symptoms. The fusiform gyrus territory has received considerable attention for its role in reading, moreover, its damage might lead to the letter-by-letter reading strategies shown by neuroanatomical investigations.^28^ Our identified biotype 2 is consistent with this investigation that though the cerebellum and fusiform gyrus do not connect physically, their functional connection (FC) contributes to reading abilities, phonological processing, and semantic memory.^29^ This abnormal FC might further influence cognitive abilities, and lead to inattention and hyperactive symptoms.

As the most prominent network in the clinical neuroscience literature regarding spontaneous intrinsic brain activity, the default mode network (DMN) underlies some of the executive function deficits, working memory deficits, and attention lapses in ADHD patients.^30^ Our results showed that the extracted common top 100 discriminative FNCs from two separate cohorts showed great overlap and correlated with multiple cognition and symptom scales. Among top 100 FNCs, the fundamental connection between the superior parietal lobule and posterior cingulate cortex, and precuneus are highly correlated with multiple cognitive abilities and symptoms, indicating that these connections from DMN regions to the superior parietal lobule are associated with several attention problems. The superior parietal lobule has been shown to play a major role in the voluntary shift of spatial visual attention and attention switching, and thus to be particularly important for regulated attentional processes and attention disengagement.^31^^,^^32^ Besides, the common connection in DMN from the precuneus to the anterior cingulate cortex is also considered a candidate locus of dysfunction in ADHD.^33^ A functional connectivity analysis suggested that structural and functional circuits linking the anterior cingulate cortex to the precuneus may represent “small-world network” long-range connections.^34^ Our results affirmed the importance of DMN in ADHD analysis and put forward a presumption that the connection between DMN and the superior parietal lobule might also be a key circuit.

Most interestingly, we illustrate the treatment record varying from 1, 2, 3, 4, 8 weeks after the treatment between the two biotypes. Though only part of the subjects received medication treatment, we still found that biotype 1 showed a steady recovery in all ADHD symptoms, and biotype 2 approached to healthy children at a smaller degree after 8 weeks. Multiple studies revealed that MPH and ATX improve attention functions and upregulate abnormal fronto-cortical activation during executive function tasks in ADHD patients.^35^ Differential DMN and cerebellar connectivity patterns between the two biotypes may influence drug responsiveness, consequently leading to varying symptom improvement outcomes. Furthermore, previous studies showed that stimulant medications like MPH are the most effective treatments for ADHD, while the non-stimulant ATX shows slightly lower but good efficacy in reducing ADHD symptoms, and they have differences side effects in the treatment of ADHD.^36^ Particularly, we found significant interaction effects between biotypes and medication in psychosomatic problems. It is interesting to note that psychosomatic problems (including headaches, stomachache, pain in various places and bowel irregularities) overlapped with multiple side effects of MPH and ATX in ADHD, suggesting that the two ADHD biotypes may show different side effects when taking medications, and these side effects mainly fall within the domain of psychosomatic problems. Further studies with more subjects could be conducted to further test this hypothesis.

Some limitations should be considered. First, ABCD is an ongoing prospective longitudinal study starting at the ages of 9–10 and includes a diverse sample enrolled at 21 research sites across the U.S. The PKU dataset is collected from a local hospital, which contains potential ADHD patients. This limitation likely reduces the similarity of the ADHD biotype patterns from two datasets. Second, only some of the ADHD patients from PKU were taking medication and have longitudinal scale information, however, the side effects and teacher reports are missing. Therefore, our analysis of medication influence is an explorative attempt. We can include more medicated ADHD patients in the future to provide a more detailed examination. Third, to validate the potential clinical utility of the identified biotypes, we used an independent dataset to track the multiple symptom domain manifestations of each biotype derived from ABCD. It is important to note that this is not a replication of our approach, but more of a validation of the biological subtypes we obtained.

Collectively, in this study, we have identified two distinct ADHD biotypes that exhibit significant group differences in functional networks and multiple cognitive abilities and symptoms, particularly in fluid intelligence and hyperactivity/impulsivity. These findings are promising for facilitating personalized medication based on biotype-guided approaches for individuals with ADHD. We also proposed a novel framework, GCN-BSD, which can jointly characterize brain imaging data and phenotypic association to build the population graph, and further use this knowledge to guide biotype detection for multiple mental disorders. Importantly, our findings indicate that the frontoparietal circuits could serve as a key signature to ADHD and provide the first evidence for the connection from the cerebellum to the fusiform gyrus to be used as a neuroimaging marker in defining the uncommon subgroup. To summarize, this study develop a more flexible deep learning-based biotype identification framework that that aims to provide support for personalized efficacy treatment of adolescent ADHD.

## Contributors

Conceptualization, AF, DZ, and JS; methodology, AF, DZ, and JS; data analysis, YF and FZ; data collection and organization, YF, LS, ZF, VDC; data analysis, AF, SY, MZ, MX, and RJ; writing—original draft preparation, AF, DZ, and YF; writing—review and editing, RJ, ZF, SY, MS, LS, VDC, and JS; visualization: AF, DZ, and JS; funding acquisition, LS, DZ, VDC, and JS; supervision, LS, VDC, and JS; data access and verification, AF, DZ, YF, LS and JS. AF, DZ, YF, LS and JS have full access to all the data in the study. All authors read and approved the final version of the manuscript and accept responsibility for the decision to submit it for publication.

## Data sharing statement

Data will be available upon reasonable request to the corresponding author (via email) after institutional approval and with a signed data access agreement or with the permission of Peking University Sixth Hospital/Institute of Mental Health. Detailed information regarding these datasets are provided in Supplementary Materials.

## Declaration of interests

The authors report no biomedical financial interests or potential conflicts of interest.
