# Supplementary material for: Functional imaging derived ADHD biotypes based on deep clustering: a study on personalized medication therapy guidance
Source: eClinicalMedicine. 2024 Oct 10;77:102876. doi: 10.1016/j.eclinm.2024.102876 (PMC11701483; doi:10.1016/j.eclinm.2024.102876)
Supplement: Supplementary [file mmc1.docx]

**Supplementary Information**

**Functional Imaging Derived ADHD Biotypes Based on Deep Clustering: A Study on Personalized Medication Therapy Guidance**

**ABCD Image acquisition**

The ABCD study was collected from 21 different sites, using Siemens Prisma, Phillips and General Electric 750 3 Tesla scanners with harmonized imaging parameters, in eyes-open condition and passive viewing of a crosshair with a repetition time of 0.8 s. The details of the ABCD study parameters were previously described ^1^. The ABCD FastTrack images with recommended active series were downloaded from the National Institute of Mental Health Data Archive (https://nda.nih.gov/).

**ABCD Quality control**

Participants from the ABCD dataset were selected based on the availability of high-quality neuroimaging data. The quality assessment of the neuroimaging data was based on the “Quality Control and Recommended Inclusion Criteria” provided as a pdf document in the “Release Notes Imaging Instruments” folder. Sample selection proceeded as follows: among 11875 individuals, 11433 participants with available resting state data passed raw quality control were included in a spatially constrained independent component analysis (ICA). Exclusions were as follows (Fig. S1):

-2153 individuals were excluded with the following criteria: not passing the Freesurfer QC; no serious MR findings; rsfMRI series passed raw QC; T1 series passed raw QC; rsfMRI number of frames > 375; fMRI B0 unwarp available; not passing fMRI manual post-processing QC; fMRI registration to T1w: less than 19; fMRI maximum dorsal cut off score: less than 65; fMRI maximum ventral cutoff score: less than 60.

-60 individuals were further excluded without good normalization of their fMRI images to the MNI standard space at baseline.

-We determined ADHD same as in previous studies ^2,3^ by past and present mental disorder diagnoses using parent-reported responses to the self-administered computerized Schedule for Affective Disorders and Schizophrenia for School-Age Children for DSM-5 (K-SADS-5).

Finally, we have 1069 ADHD participants included, and 1164 demographically matched HCs were included in our study.

**Table S1.** Demographic information and symptom scale of the subjects from ABCD.

| **Variables** | **HC(n=1164)** | **ADHD(n=1069)** | **P-value** |
| --- | --- | --- | --- |
| **Demographic variables** | | | |
| Age(months) | 119$\pm$7 | 119$\pm$7 | 0.76 |
| Gender(M/F) | 760/404 | 697/363 | 0.96 |
| **Cognitive Scales** | | | |
| Picture Vocabulary Test | 107.45±16.44 | 105.34±16.26 | 3.43e-4 |
| Flanker Inhibitory Control and Attention Test | 96.54±14.28 | 94.53±13.76 | 1.82e-5 |
| List Sorting Working Memory | 101.66±14.73 | 98.15±14.50 | 2.09e-13 |
| Dimensional Change Card Sort Test | 98.23±15.65 | 94.01±14.32 | 1.36e-14 |
| Pattern Comparison Processing Speed Test | 95.18±21.30 | 91.06±22.86 | 7.10e-11 |
| Picture Sequence Memory Test | 102.82±16.67 | 98.37±16.60 | 5.64e-15 |
| Oral Reading Recognition Test | 103.88±19.90 | 99.28±18.63 | 1.52e-14 |
| Fluid Cognition | 97.31±16.22 | 92.88±17.72 | 1.64e-25 |
| Crystallized Cognition | 106.84±17.27 | 102.15±17.39 | 1.17e-11 |
| Total cognition | 102.33±17.54 | 96.37±17.60 | 6.91e-25 |


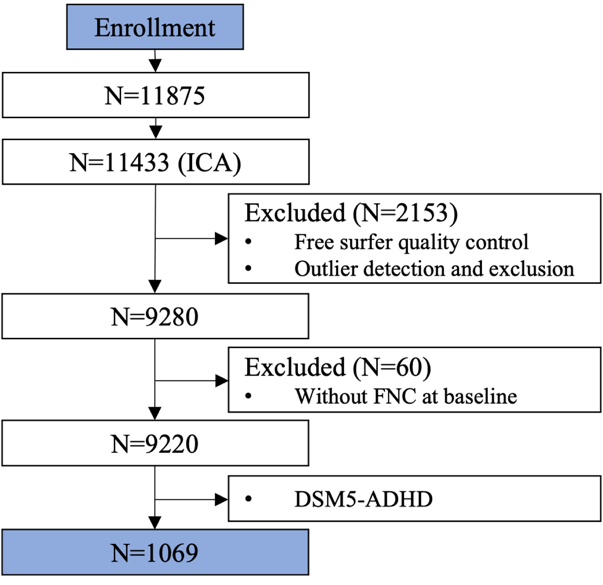


**Figure S1.** Flowchart of sample selection. Data quality for resting-state fMRI.

**PKU Image acquisition**

All participants met the following criteria: (1) right-handed, (2) no history of head trauma with a loss of consciousness, (3) no history of neurological disorders or other severe disease, (4) no current diagnosis of major depressive disorder (MDD), schizophrenia, clinically significant panic disorder, bipolar disorder, pervasive developmental disorders, or mental retardation, (5) no excessive head movements (>3.0 mm of translation or degrees of rotation in any direction), and (6) a full-scale intelligence quotient (IQ) above 80. Furthermore, participants with any history of psychiatric disorders were also excluded as HCs. Verbal IQ, performance IQ, and full-scale IQ were measured by the Wechsler Child/Adult Intelligence Scale, Third Edition.

The severity of inattentive symptoms, hyperactive/impulsive symptoms, and total ADHD symptoms of all subjects was evaluated by the ADHD Rating Scale-IV (ADHD RS-IV), rating one-four (“never” is rated as 1, “occasionally” is 2; “often” is 3; “always” is 4). This scale contains nine inattentive and nine hyperactive/impulsive symptoms of ADHD described in the DSM-IV. The higher the scores were, the more serious the ADHD symptoms were.

Besides, the Conners’ Parent Rating Scale (CPRS) was used to assess the hyperactive/impulsive symptoms in child participants with ADHD. The CPRS is a widely used instrument for screening and evaluating ADHD-related symptoms as well as other behavioral problems frequently associated with ADHD in children. It contains 48 items and can be divided into six factors: conduct problems, learning problems, psychosomatic problems, impulsivity–hyperactivity, anxiety, and ADHD index. The parents rate each item using a 4-point Likert-type scale (“never/seldom” is rated as 0, “sometimes” is 1; “quite often” is 2, and “very often” is 3). The higher the score, the more severe the corresponding problem is.

**Table S2.** Demographic information and symptom scale of the subjects from PKU.

| **Variables** | **HC(n=105)** | **ADHD(n=130)** | **P-value** |
| --- | --- | --- | --- |
| **Demographic variables** | | | |
| Age(years) | 9.71±2.02 | 9.73±2.0$1$ | 0.93 |
| Gender(M/F) | 66/39 | 91/39 | 0.25 |
| **RS-IV Symptom Scales** | | | |
| Inattention | 18.58±5.30 | $2$6.54±4.0$3$ | 3.00e-4 |
| Impulsivity–Hyperactivity | 14.83±4.91 | 19.77±5.53 | 5.50e-3 |
| Total | 33.42±9.39 | 46.31±6.87 | 6.00e-4 |
| **Wechsler Child/Adult Intelligence Scale** | | | |
| VIQ | 118.00±7.87 | 112.47±16.68 | 0.16 |
| PIQ | 106.67±9.40 | 105.30±14.47 | 0.75 |
| TIQ | 114.50±7.01 | 106.60±13.44 | 0.04 |
| **CPRS** **Symptom Scales** | | | |
| Conduct Problems | 4.33±5.24 | 12.98±6.59 | 8.51e-3 |
| Learning Problems | 1.67±2.25 | 7.57±2.32 | 1.00e-3 |
| Psychosomatic Problems | 0.67±1.03 | 1.55±1.82 | 0.09 |
| Impulsivity–Hyperactivity | 1.00±1.55 | 5.41±2.52 | 5.01e-4 |
| Anxiety | 1.83±3.12 | 2.86±2.22 | 0.46 |
| ADHD index | 2.83±3.34 | 13.71±4.46 | 3.18e-4 |

**Table S3. Conners’ Parent Rating Scale Items**

| Items |
| --- |
| 1. Tears things (including nails, fingers, hair, clothes, etc.) |
| 2. Displays defiance toward adults, with reckless speech and behavior. |
| 3. Has difficulty getting along with peers or classmates. |
| 4. Prone to impulsive behavior. |
| 5. Exhibits controlling or manipulative tendencies when performing tasks. |
| 6. Sucking or chewing (thumbs, clothes, blankets, etc.) . |
| 7. Prone to frequent crying. |
| 8. Easily provoked to anger. |
| 9. Displays a tendency towards daydreaming. |
| 10. Experiences learning difficulties. |
| 11. Frequently expresses feelings of restlessness or unease. |
| 12. Anxious in new places, with new people, or when going to school. |
| 13. Hyperactive or overly energetic. |
| 14. Exhibits destructive tendencies. |
| 15. Tendency to fabricate stories or engage in dishonesty. |
| 16. Shy or socially withdrawn. |
| 17. More accident-prone compared to peers. |
| 18. Displays weaker language skills compared to peers (e.g., baby talk, stuttering, difficult to understand) . |
| 19. Avoids accepting responsibility for mistakes or shifts blame to others. |
| 20. Argumentative or confrontational behavior. |
| 21. Frequently sulks or pouts. |
| 22. Occasionally takes money or belongings from parents or others without permission. |
| 23. Non-compliant with instructions from teachers and parents, or frequently complains when complying. |
| 24. Exhibits heightened fear of isolation, illness, or death compared to peers. |
| 25. Struggles to follow through and complete tasks. |
| 26. Easily experiences emotional hurt or sensitivity. |
| 27. Displays bullying behavior, targeting weaker individuals. |
| 28. Engages in repetitive actions. |
| 29. Demonstrates cruelty in behavior. |
| 30. Exhibits immature behavior, requiring assistance with tasks they are capable of and seeking constant reassurance from adults. |
| 31. Easily distracted, with a short attention span. |
| 32. Reports frequent headaches. |
| 33. Displays rapid and intense mood swings. |
| 34. Disregards rules or shows aversion to restrictions. |
| 35. Frequently engages in physical altercations. |
| 36. Struggles to maintain positive relationships with siblings. |
| 37. Easily frustrated when faced with challenges. |
| 38. Disrupts or distracts other children. |
| 39. Displays persistent unhappiness. |
| 40. Experiences eating difficulties (e.g., poor appetite, leaving the table during meals) . |
| 41. Stomachaches. |
| 42. Sleep problems (difficulty falling asleep, waking up too early or during the night) . |
| 43. Frequently feels pain in various places. |
| 44. Exhibits frequent vomiting or nausea. |
| 45. Feels consistently deceived or mistrusted within the household. |
| 46. Engages in self-aggrandizing or exaggerated boasting. |
| 47. Frequently imagines being threatened or in danger. |
| 48. Experiences bowel irregularities (e.g., frequent diarrhea, irregular bowel movements, constipation) . |

**Table S4. Conners’ Parent Rating Scale factors**

| CPRS Factors | Item No. |
| --- | --- |
| Conduct Problems | 2, 8, 14, 19, 20, 21, 22, 23, 27, 33, 34, 39 |
| Learning Problems | 10, 25, 31, 37 |
| Psychosomatic Problems | 32, 41, 43, 44, 48 |
| Impulsivity-Hyperactivity | 4, 5, 11, 13 |
| Anxiety | 12, 16, 24, 47 |
| ADHD Index (hyperactivity index) | 4, 7, 11, 13, 14, 25, 31, 33, 37, 38 |

**Medication information**

The full name of methylphenidate is methylphenidate hydrochloride extended-release tablets. The chemical name is α-phenyl−2-piperidilacetic acid methyl ester hydrochloride, and the brand name is Concerta (Janssen Pharmaceuticals, Inc.; http://www.xian-janssen.com.cn). The starting dose of methylphenidate was usually 18 mg/day, and the dose was modified every 7 days based on efficacy and side effects by increasing or decreasing by 18 mg/day. The maximum dose was 54 mg/day.

Atomoxetine is divided into atomoxetine hydrochloride capsules and atomoxetine hydrochloride oral solution, and the chemical name is (-)-N-methyl−3-(o-tolyloxy)−3-phenylpropylamine. The brand name of atomoxetine hydrochloride capsules is Strattera (LILLY S.A.; https://www.lillymedical.cn/zh-cn/cns/Strattera). The starting dose of Strattera was typically 0.5 mg/day, and the dose was modified every 7 days based on efficacy and side effects. The maximum daily dose did not exceed 1.4 mg/kg or 100 mg. Atomoxetine hydrochloride oral solution was administered to patients with dysphagia (1 patient in this study) when taking the capsules, and the dose was converted based on 4 mg/mL.

44 patients from PKU received medical treatment, 17 of them were treated with atomoxetine (ATX) and 27 of them were treated with methylphenidate (MPH). At the week 8 follow-up, the mean dose of methylphenidate in the treatment group was (23.26 ± 8.49) mg/day and the dose-to-weight ratio was (0.67 ± 0.19) mg/kg.d; the mean dose of atomoxetine in the treatment group was (31.25 ± 11.71) mg/day and the dose-to-weight ratio was (0.89 ± 0.23) mg/kg.d.

**Table S5.** Medication information of the subjects under medication from PKU.

| **Variables** | **MPH (n=27)** | **ATX(n=17)** |
| --- | --- | --- |
| Mean dose(mg/day) | 23.26 ± 8.49 | 31.25 ± 11.71 |
| Dose-to-weight ratio(mg/kg) | 0.67 ± 0.19 | 0.89 ± 0.23 |

We recorded their symptom scales at baseline, week 1, week 2, week 3, week 4, and week 8. Demographic information was shown in Table S6.

**Table S6.** **Symptom changes of the ADHD patients under two medications at PKU.**

| **Medications** |  | **MPH (n=27)** | **ATX(n=17)** | **P-value** |
| --- | --- | --- | --- | --- |
| **Demographics** | Age(years) | 12.26±1.76 | 9.53$\pm$1.73 | 1.44e-5 |
|  | Gender(M/F) | 18/9 | 14/3 | 0.25 |
| **Baseline** | RS-inattention | 27.07±2.96 | 28.76±2.63 | 0.06 |
|  | RS-IH | 19.59±6.08 | 21.35±5.24 | 0.31 |
|  | RS-total | 46.67±8.25 | 50.12±6.71 | 0.14 |
|  | CPRS-Conduct Problems | 13.69±6.60 | 11.23±4.43 | 0.15 |
|  | CPRS-Learning Problems | 7.04±2.33 | 7.64±2.20 | 0.40 |
|  | CPRS-Psychosomatic Problems | 1.81±1.84 | 2.18±2.06 | 0.56 |
|  | CPRS-Impulsivity–Hyperactivity | 5.59±3.04 | 6.70±3.07 | 0.25 |
|  | CPRS-Anxiety | 2.26±1.72 | 2.06±1.88 | 0.72 |
|  | CPRS-ADHD index | 14.20±5.18 | 15.12±4.20 | 0.53 |
| **Week 1** | RS-inattention | 21.79±5.24 | 24.19±4.91 | 0.13 |
|  | RS-IH | 15.30±4.18 | 18.85±5.97 | 0.04 |
|  | RS-total | 37.10±8.09 | 43.05±10.16 | 0.05 |
|  | CPRS-Conduct Problems | 10.85±5.93 | 11.11±5.36 | 0.88 |
|  | CPRS-Learning Problems | 5.88±2.13 | 7.26±2.15 | 0.05 |
|  | CPRS-Psychosomatic Problems | 1.17±1.68 | 1.61±1.69 | 0.41 |
|  | CPRS-Impulsivity–Hyperactivity | 3.60±2.72 | 4.88±2.88 | 0.15 |
|  | CPRS-Anxiety | 1.74±1.67 | 2.23±1.82 | 0.37 |
|  | CPRS-ADHD index | 9.99±4.79 | 12.29±4.21 | 0.10 |
| **Week 2** | RS-inattention | 21.74±4.76 | 24.00±4.87 | 0.14 |
|  | RS-IH | 14.85±4.18 | 18.70±5.17 | 0.02 |
|  | RS-total | 36.59±8.10 | 42.71±8.61 | 0.03 |
|  | CPRS-Conduct Problems | 10.32±5.22 | 11.52±5.49 | 0.48 |
|  | CPRS-Learning Problems | 5.56±2.02 | 6.77±2.61 | 0.12 |
|  | CPRS-Psychosomatic Problems | 1.30±1.58 | 2.09±2.03 | 0.18 |
|  | CPRS-Impulsivity–Hyperactivity | 3.60±2.44 | 4.89±2.64 | 0.11 |
|  | CPRS-Anxiety | 1.96±1.53 | 1.82±1.42 | 0.76 |
|  | CPRS-ADHD index | 9.06±4.20 | 12.25±4.24 | 0.02 |
| **Week 3** | RS-inattention | 20.47±4.68 | 22.76±5.41 | 0.16 |
|  | RS-IH | 14.88±4.36 | 18.35±5.23 | 0.03 |
|  | RS-total | 35.36±8.07 | 41.11±9.19 | 0.04 |
|  | CPRS-Conduct Problems | 9.59±5.75 | 10.18±5.23 | 0.73 |
|  | CPRS-Learning Problems | 5.29±2.46 | 6.41±2.45 | 0.15 |
|  | CPRS-Psychosomatic Problems | 1.05±1.43 | 1.76±1.92 | 0.20 |
|  | CPRS-Impulsivity–Hyperactivity | 3.33±2.11 | 4.76±2.38 | 0.05 |
|  | CPRS-Anxiety | 1.54±1.71 | 1.65±1.58 | 0.83 |
|  | CPRS-ADHD index | 8.65±4.40 | 11.35±3.79 | 0.04 |
| **Week 4** | RS-inattention | 19.33±5.68 | 23.00±4.91 | 0.03 |
|  | RS-IH | 14.62±4.97 | 17.88±5.51 | 0.06 |
|  | RS-total | 33.96±9.86 | 40.88±9.43 | 0.03 |
|  | CPRS-Conduct Problems | 9.78±6.47 | 10.76±4.51 | 0.55 |
|  | CPRS-Learning Problems | 5.00±2.38 | 6.82±2.53 | 0.02 |
|  | CPRS-Psychosomatic Problems | 1.33±1.94 | 1.88±1.58 | 0.31 |
|  | CPRS-Impulsivity–Hyperactivity | 3.11±2.21 | 4.94±2.63 | 0.02 |
|  | CPRS-Anxiety | 2.07±1.73 | 1.94±1.48 | 0.78 |
|  | CPRS-ADHD index | 8.74±4.19 | 12.12±3.39 | 0.01 |
| **Week 8** | RS-inattention | 19.07±6.70 | 21.06±6.73 | 0.36 |
|  | RS-IH | 14.18±5.62 | 17.00±6.29 | 0.14 |
|  | RS-total | 33.25±11.68 | 38.32±12.12 | 0.21 |
|  | CPRS-Conduct Problems | 10.40±7.08 | 9.47±5.33 | 0.62 |
|  | CPRS-Learning Problems | 5.00±2.84 | 5.53±2.40 | 0.51 |
|  | CPRS-Psychosomatic Problems | 0.96±1.70 | 1.12±1.32 | 0.74 |
|  | CPRS-Impulsivity–Hyperactivity | 3.30±2.25 | 4.24±2.22 | 0.19 |
|  | CPRS-Anxiety | 1.63±1.52 | 1.65±1.93 | 0.97 |
|  | CPRS-ADHD index | 8.74±5.01 | 10.41±4.00 | 0.23 |

**Table S7. Raw scale reduction rate of the subjects under medication from PKU.**

| **Medications** |  | **MPH (n=27)** | **ATX(n=17)** | **P-value** |
| --- | --- | --- | --- | --- |
| **Week 1** | RS-inattention | 0.19±0.18 | 0.16±0.16 | 0.60 |
|  | RS-IH | 0.16±0.22 | 0.13±0.24 | 0.69 |
|  | RS-total | 0.19±0.16 | 0.16±0.16 | 0.57 |
|  | CPRS-Conduct Problems | 0.16±0.17 | 0.16±0.20 | 0.27 |
|  | CPRS-Learning Problems | 0.16±0.17 | 0.16±0.20 | 0.39 |
|  | CPRS-Psychosomatic Problems | 0.17±0.17 | 0.19±0.18 | 0.64 |
|  | CPRS-Impulsivity–Hyperactivity | 0.16±0.18 | 0.16±0.20 | 0.21 |
|  | CPRS-Anxiety | 0.16±0.18 | 0.15±0.19 | 0.59 |
|  | CPRS-ADHD index | 0.16±0.17 | 0.16±0.20 | 0.20 |
| **Week 2** | RS-inattention | 0.20±0.17 | 0.16±0.18 | 0.38 |
|  | RS-IH | 0.16±0.22 | 0.15±0.18 | 0.90 |
|  | RS-total | 0.19±0.16 | 0.17±0.23 | 0.58 |
|  | CPRS-Conduct Problems | 0.16±0.17 | 0.19±0.18 | 0.13 |
|  | CPRS-Learning Problems | 0.17±0.17 | 0.19±0.18 | 0.40 |
|  | CPRS-Psychosomatic Problems | 0.17±0.17 | 0.21±0.18 | 0.30 |
|  | CPRS-Impulsivity–Hyperactivity | 0.17±0.17 | 0.19±0.18 | 0.90 |
|  | CPRS-Anxiety | 0.17±0.17 | 0.18±0.18 | 0.49 |
|  | CPRS-ADHD index | 0.17±0.17 | 0.19±0.18 | 0.07 |
| **Week 3** | RS-inattention | 0.25±0.19 | 0.19±0.18 | 0.29 |
|  | RS-IH | 0.17±0.26 | 0.17±0.20 | 0.94 |
|  | RS-total | 0.22±0.19 | 0.19±0.16 | 0.49 |
|  | CPRS-Conduct Problems | 0.20±0.17 | 0.20±0.19 | 0.11 |
|  | CPRS-Learning Problems | 0.20±0.17 | 0.20±0.19 | 0.21 |
|  | CPRS-Psychosomatic Problems | 0.19±0.17 | 0.25±0.18 | 0.15 |
|  | CPRS-Impulsivity–Hyperactivity | 0.20±0.18 | 0.20±0.19 | 0.27 |
|  | CPRS-Anxiety | 0.21±0.18 | 0.19±0.19 | 0.88 |
|  | CPRS-ADHD index | 0.20±0.17 | 0.20±0.19 | 0.06 |
| **Week 4** | RS-inattention | 0.28±0.23 | 0.20±0.16 | 0.13 |
|  | RS-IH | 0.23±0.30 | 0.15±0.21 | 0.34 |
|  | RS-total | 0.27±0.23 | 0.18±0.15 | 0.14 |
|  | CPRS-Conduct Problems | 0.20±0.17 | 0.20±0.20 | 0.06 |
|  | CPRS-Learning Problems | 0.20±0.17 | 0.20±0.20 | 0.08 |
|  | CPRS-Psychosomatic Problems | 0.20±0.17 | 0.23±0.19 | 0.59 |
|  | CPRS-Impulsivity–Hyperactivity | 0.20±0.17 | 0.20±0.20 | 0.11 |
|  | CPRS-Anxiety | 0.20±0.17 | 0.17±0.19 | 0.50 |
|  | CPRS-ADHD index | 0.20±0.17 | 0.20±0.20 | 0.02 |
| **Week 8** | RS-inattention | 0.35±0.29 | 0.18±0.14 | 0.01 |
|  | RS-IH | 0.29±0$.34$ | 0.15±0.19 | 0.11 |
|  | RS-total | 0.33±0.30 | 0.17±0.13 | 0.02 |
|  | CPRS-Conduct Problems | 0.21±0.22 | 0.24±0.24 | 0.55 |
|  | CPRS-Learning Problems | 0.21±0.22 | 0.24±0.24 | 0.57 |
|  | CPRS-Psychosomatic Problems | 0.21±0.23 | 0.23±0.24 | 0.41 |
|  | CPRS-Impulsivity–Hyperactivity | 0.21±0.22 | 0.24±0.24 | 0.24 |
|  | CPRS-Anxiety | 0.21±0.22 | 0.22±0.24 | 0.62 |
|  | CPRS-ADHD index | 0.21±0.22 | 0.24±0.24 | 0.23 |

**Table S8. Scale reduction rate of the subjects under medication from PKU.**

| **Medications** |  | **MPH (n=27)** | **ATX(n=17)** | **P-value** |
| --- | --- | --- | --- | --- |
| **Week 1** | RS-inattention | 0.16±0.17 | 0.11±0.17 | 0.32 |
|  | RS-IH | 0.13±0.15 | 0.01±0.24 | 0.07 |
|  | RS-total | 0.16±0.13 | 0.08±0.19 | 0.13 |
|  | CPRS-Conduct Problems | -0.08±0.63 | -0.28±1.05 | 0.48 |
|  | CPRS-Learning Problems | -0.04±0.37 | -0.23±0.32 | 0.08 |
|  | CPRS-Psychosomatic Problems | 0.07±0.72 | 0.01±0.67 | 0.47 |
|  | CPRS-Impulsivity–Hyperactivity | 0.22±0.43 | 0.23±1.03 | 0.10 |
|  | CPRS-Anxiety | -0.10±1.22 | 0.23±0.65 | 0.65 |
|  | CPRS-ADHD index | 0.17±0.30 | 0.03±0.39 | 0.09 |
| **Week 2** | RS-inattention | 0.14±0.16 | 0.08±0.17 | 0.27 |
|  | RS-IH | 0.15±0.15 | 0.002±0.23 | 0.02 |
|  | RS-total | 0.16±0.13 | 0.06±0.18 | 0.06 |
|  | CPRS-Conduct Problems | -0.07±0.52 | -0.25±0.58 | 0.31 |
|  | CPRS-Learning Problems | 0.07±0.30 | -0.09±0.40 | 0.15 |
|  | CPRS-Psychosomatic Problems | 0.004±0.82 | -0.43±1.07 | 0.20 |
|  | CPRS-Impulsivity–Hyperactivity | 0.001±0.92 | -0.14±0.75 | 0.60 |
|  | CPRS-Anxiety | -0.28±1.04 | -0.06±0.57 | 0.38 |
|  | CPRS-ADHD index | 0.28±0.32 | 0.04±0.36 | 0.03 |
| **Week 3** | RS-inattention | 0.14±0.16 | 0.07±0.18 | 0.18 |
|  | RS-IH | 0.16±0.18 | 0.02±0.23 | 0.05 |
|  | RS-total | 0.18±0.15 | 0.08±0.19 | 0.09 |
|  | CPRS-Conduct Problems | 0.18±0.44 | -0.007±0.49 | 0.20 |
|  | CPRS-Learning Problems | 0.20±0.29 | 0.04±0.34 | 0.12 |
|  | CPRS-Psychosomatic Problems | 0.25±0.63 | -0.21±0.72 | 0.06 |
|  | CPRS-Impulsivity–Hyperactivity | 0.21±0.50 | -0.10±0.68 | 0.11 |
|  | CPRS-Anxiety | 0.01±1.23 | -0.02±0.79 | 0.92 |
|  | CPRS-ADHD index | 0.35±0.28 | 0.14±0.35 | 0.04 |
| **Week 4** | RS-inattention | 0.22±0.21 | 0.11±0.17 | 0.06 |
|  | RS-IH | 0.20±0.24 | 0.07±0.26 | 0.11 |
|  | RS-total | 0.24±0.20 | 0.14±0.20 | 0.08 |
|  | CPRS-Conduct Problems | 0.15±0.46 | -0.13±0.59 | 0.10 |
|  | CPRS-Learning Problems | 0.24±0.34 | 0.01±0.34 | 0.04 |
|  | CPRS-Psychosomatic Problems | -0.05±1.21 | -0.34±0.92 | 0.42 |
|  | CPRS-Impulsivity–Hyperactivity | 0.28±0.48 | -0.17±0.74 | 0.04 |
|  | CPRS-Anxiety | -0.31±1.22 | -0.10±0.54 | 0.45 |
|  | CPRS-ADHD index | 0.34±0.30 | 0.08±0.32 | 0.01 |
| **Week 8** | RS-inattention | 0.16±0.25 | 0.08±0.23 | 0.33 |
|  | RS-IH | 0.24±0$.$27 | 0.13±0.31 | 0.24 |
|  | RS-total | 0.25±0.25 | 0.18±0.26 | 0.37 |
|  | CPRS-Conduct Problems | 0.02±0.66 | -0.003±0.62 | 0.90 |
|  | CPRS-Learning Problems | 0.26±0.37 | 0.17±0.39 | 0.45 |
|  | CPRS-Psychosomatic Problems | 0.26±0.83 | -0.07±0.65 | 0.20 |
|  | CPRS-Impulsivity–Hyperactivity | 0.17±0.49 | -0.21±0.66 | 0.05 |
|  | CPRS-Anxiety | 0.10±0.89 | -0.004±0.74 | 0.67 |
|  | CPRS-ADHD index | 0.33±0.35 | 0.17±0.37 | 0.16 |

**Data preprocessing**

The FastTrack images are unprocessed imaging data that completed and passed raw quality control (QC). The resting-state functional magnetic resonance imaging (rsfMRI) data were preprocessed with a combination of FMRIB Software Library v6.0 (FSL) toolbox and Statistical Parametric Mapping 12 (SPM) toolbox under the MATLAB 2019b environment. First, we performed the rigid body motion correction with the *mcflirt* tool in FSL before the distortion correction. Then, the distortion in the fMRI volume was corrected using the *applytopup* tool in FSL based on field map coefficients obtained with the top-up tool in FSL. After distortion correction, we discarded 10 initial scans with large signal changes to allow the tissue to reach a steady state of radiofrequency excitation. Next, the fMRI data were warped into the standard Montreal Neurological Institute (MNI) space based on the echo-planar imaging (EPI) template, resampling to 3×3×3 mm3 isotropic voxels using the normalization tool in SPM. Finally, the resliced fMRI images were subsequently smoothed with 6 mm full width at half maximum Gaussian kernel.

**NeuroMark framework**

The NeuroMark framework leverages an adaptive-ICA technique that automates the estimation of reproducible functional brain markers across subjects, datasets, and studies ^4^. After preprocessing, the data were decomposed into 53 resting-state networks (RSN) by utilizing a spatially constrained ICA using the spatial network templates from the NeuroMark, which were derived from two large-sample healthy control datasets (1828 healthy subjects in total). We utilized the Neuromark_fMRI_1.0 priors resulting in 53 subject-specific independent components (ICs) and their corresponding time courses (TCs). Four additional post-processing steps were performed to carefully regress out the remaining noise in the TCs: 1) detrending linear, quadratic, and cubic trends; 2) removal of detected outliers; 3) multiple regression of the head motions parameters (3 rotations and 3 translations) and their derivatives; 4) band-pass filtering with a cutoff frequency of 0.01 Hz-0.15 Hz. The NeuroMark template contains fifty-three independent components which were identified as meaningful intrinsic connectivity networks (ICNs) and arranged into 7 functional networks according to the anatomic and functional prior knowledge, including 4 cerebellar (CB), 19 cognitive control (CC), 2 auditory (AU), 7 default mode (DM), 5 subcortical (SC), 9 somatomotor (SM), and 9 visual (VI) components (Table S9 and Fig. S2).

**Table S9. Peak coordinates of FNC nodes of interest.**

| ICN (IC index) | X | Y | Z | Domain |
| --- | --- | --- | --- | --- |
| Caudate (69) | 6.5 | 10.5 | 5.5 | **Subcortical** |
| Subthalamus/hypothalamus (53) | -2.5 | -13.5 | -1.5 |  |
| Putamen (98) | -26.5 | 1.5 | -0.5 |  |
| Caudate (99) | 21.5 | 10.5 | -3.5 |  |
| Thalamus (45) | -12.5 | -18.5 | 11.5 |  |
| Superior temporal gyrus ([STG], 21) | 62.5 | -22.5 | 7.5 | **Auditory** |
| Middle temporal gyrus ([MTG], 56) | -42.5 | -6.5 | 10.5 |  |
| Postcentral gyrus ([PoCG], 3) | 56.5 | -4.5 | 28.5 | **Sensorimotor** |
| Left postcentral gyrus ([L PoCG], 9) | -38.5 | -22.5 | 56.5 |  |
| Paracentral lobule ([ParaCL], 2) | 0.5 | -22.5 | 65.5 |  |
| Right postcentral gyrus ([R PoCG], 11) | 38.5 | -19.5 | 55.5 |  |
| Superior parietal lobule ([SPL], 27) | -18.5 | -43.5 | 65.5 |  |
| Paracentral lobule ([ParaCL], 54) | -18.5 | -9.5 | 56.5 |  |
| Precentral gyrus ([PreCG], 66) | -42.5 | -7.5 | 46.5 |  |
| Superior parietal lobule ([SPL], 80) | 20.5 | -63.5 | 58.5 |  |
| Postcentral gyrus ([PoCG], 72) | -47.5 | -27.5 | 43.5 |  |
| Calcarine gyrus ([CalcarineG], 16) | -12.5 | -66.5 | 8.5 | **Visual** |
| Middle occipital gyrus ([MOG], 5) | -23.5 | -93.5 | -0.5 |  |
| Middle temporal gyrus ([MTG], 62) | **48.5** | **-60.5** | **10.5** |  |
| Cuneus (15) | 15.5 | -91.5 | 22.5 |  |
| Right middle occipital gyrus ([R MOG], 12) | **38.5** | **-73.5** | **6.5** |  |
| Fusiform gyrus (93) | 29.5 | -42.5 | -12.5 |  |
| Inferior occipital gyrus ([IOG], 20) | **-36.5** | **-76.5** | **-4.5** |  |
| Lingual gyrus ([LingualG], 8) | -8.5 | -81.5 | -4.5 |  |
| Middle temporal gyrus ([MTG], 77) | **-44.5** | **-57.5** | **-7.5** |  |
| Inferior parietal lobule ([IPL], 68) | 45.5 | -61.5 | 43.5 | **Cognitive Control** |
| Insula (33) | -30.5 | 22.5 | -3.5 |  |
| Superior medial frontal gyrus ([SMFG], 43) | **-0.5** | **50.5** | **29.5** |  |
| Inferior frontal gyrus ([IFG], 70) | -48.5 | 34.5 | -0.5 |  |
| Right inferior frontal gyrus ([R IFG], 61) | 53.5 | 22.5 | 13.5 |  |
| Middle frontal gyrus ([MiFG], 55) | -41.5 | 19.5 | 26.5 |  |
| Inferior parietal lobule ([IPL], 63) | -53.5 | -49.5 | 43.5 |  |
| Left inferior parietal lobue ([R IPL], 79) | 44.5 | -34.5 | 46.5 |  |
| Supplementary motor area ([SMA], 84) | -6.5 | 13.5 | 64.5 |  |
| Superior frontal gyrus ([SFG], 96) | -24.5 | 26.5 | 49.5 |  |
| Middle frontal gyrus ([MiFG], 88) | 30.5 | 41.5 | 28.5 |  |
| Hippocampus ([HiPP], 48) | 23.5 | -9.5 | -16.5 |  |
| Left inferior parietal lobue ([L IPL], 81) | 45.5 | -61.5 | 43.5 |  |
| Middle cingulate cortex ([MCC], 37) | -15.5 | 20.5 | 37.5 |  |
| Inferior frontal gyrus ([IFG], 67) | 39.5 | 44.5 | -0.5 |  |
| Middle frontal gyrus ([MiFG], 38) | -26.5 | 47.5 | 5.5 |  |
| Hippocampus ([HiPP], 83) | -24.5 | -36.5 | 1.5 |  |
| Precuneus (32) | -8.5 | -66.5 | 35.5 | **Default mode** |
| Precuneus (40) | -12.5 | -54.5 | 14.5 |  |
| Anterior cingulate cortex ([ACC], 23) | **-2.5** | **35.5** | **2.5** |  |
| Posterior cingulate cortex ([PCC], 71) | -5.5 | -28.5 | 26.5 |  |
| Anterior cingulate cortex ([ACC], 17) | -9.5 | 46.5 | -10.5 |  |
| Precuneus (51) | -0.5 | -48.5 | 49.5 |  |
| Posterior cingulate cortex ([PCC], 94) | -2.5 | 54.5 | 31.5 |  |
| Cerebellum ([CB], 13) | -30.5 | -54.5 | -42.5 | **Cerebellum** |
| Cerebellum ([CB], 18) | -32.5 | -79.5 | -37.5 |  |
| Cerebellum ([CB], 4) | 20.5 | -48.5 | -40.5 |  |
| Cerebellum ([CB], 7) | 30.5 | -63.5 | -40.5 |  |


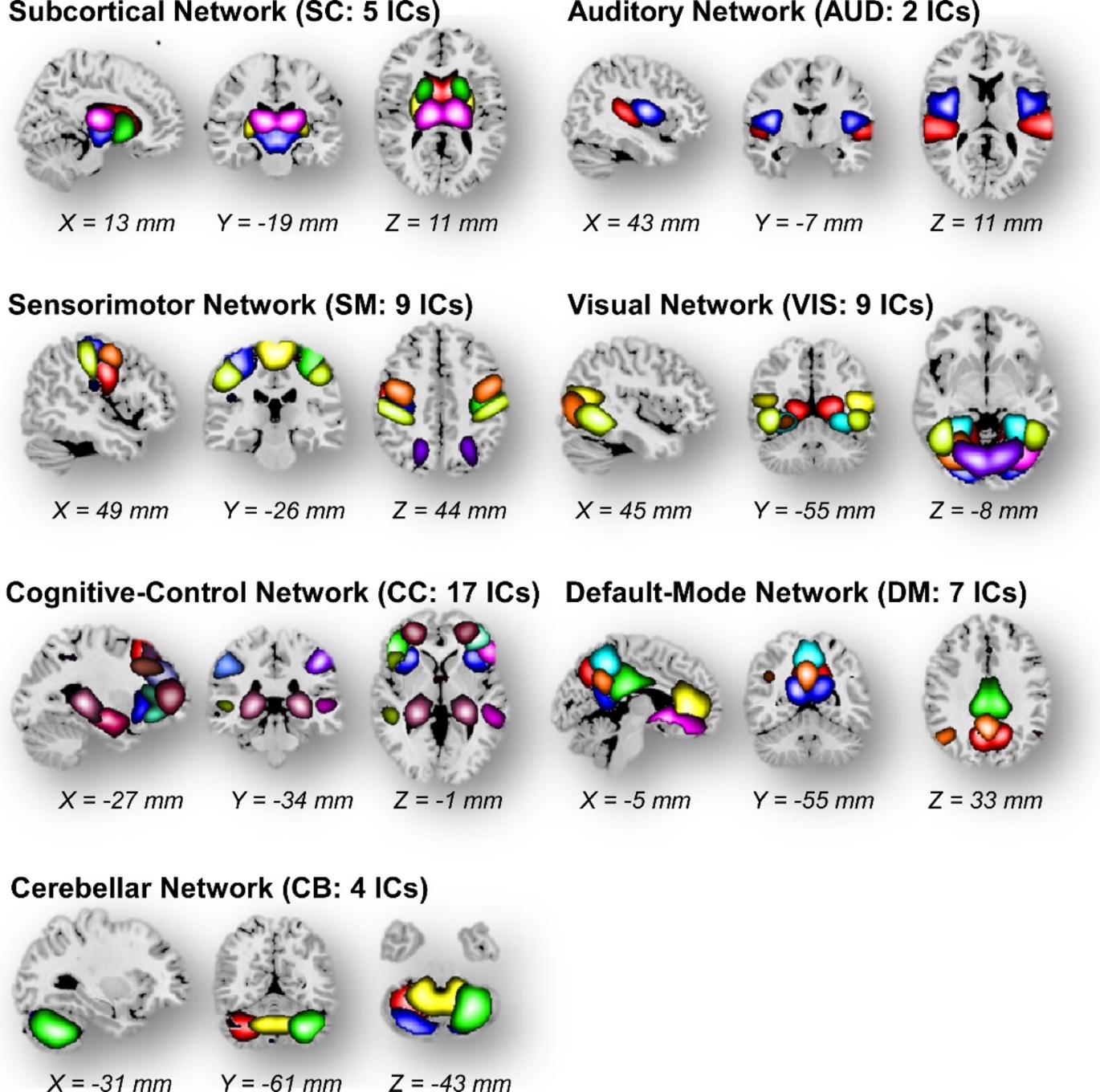


**Figure S2.** Spatial maps of the 53 independent components (ICs) are arranged into 7 functional networks

according to the anatomic and functional prior knowledge ^4^.

**Graph Convolution Network for Biological Subtype Detection (GCN-BSD)**

**Overview**

As shown in **Fig. 1**, GCN-BSD is an autoencoder-based GCN model. The proposed architecture consists of three main elements: an encoder, an embedding layer, and a decoder. The encoder accounts for abstracting the input features into low-resolution embeddings with the adjacency matrix. By transforming the embeddings, the decoder reconstructs the features.

The encoder and decoder are both built upon the GCN module, as the blocks in **Fig. 1**. The encoder and decoder are connected via the embeddings. By putting the embeddings into the fully connected layer, we adopted the binary cross entropy function to learn group-discriminative features, which identified the subject labels as patients or healthy controls for all subjects. Besides, we put the patient subgraph with the connection unchanged into the clustering layer for subtype detection. The deep K-Means loss function is adopted as the clustering loss. To better extract high-resolution embeddings, we also adopted the reconstruction loss. The GCN-BSD can then be formulated as minimizing the following function:

$L= \sum\gamma_{1}L_{rec}(X)+\gamma_{2}L_{CE}(X)+\gamma_{3}L_{cluster}(X)$ (1)

where $\gamma_{1}, \gamma_{2}, and \gamma_{3}$ regulate the trade-off among seeking good representation for $X$, which is set for subtype clustering, meanwhile is faithful to the original feature and maintains label information.

**GCN**

Graph $G=\left( V, E \right)$ is defined as a set of nodes $V=\left( v_{1}, \ldots,v_{n} \right)$, and edges $E$ ^5^. Given FNC features as input, the node feature is a vector $X\in R^{\left| V \right|\times d}$. The adjacency matrix A is defined entry-wise, where $A_{i_{1}i_{2}}=w_{i_{1}i_{2}}$ if $\left( i_{1}i_{2} \right)\in E$ and $A_{i_{1}i_{2}}=0$ if $\left( i_{1}i_{2} \right)\notin E$, for every pair $\left( i_{1}i_{2} \right)\in V$. The degree matrix $D$ is a diagonal matrix with $D_{ii}$ being the degree of the $i$th node. In 2017 Kipf et al. first proposed GCN, where they applied Flourier transform and Taylor’s expansion formula in graph neural network and introduced spectral graph convolution theory. The spectral analysis of the graph signals relies on the graph Laplacian matrix, which can be defined as $L=D-A$, and symmetric normalization is defined as $L=D^{-\frac{1}{2}}\left( D-A \right)D^{-\frac{1}{2}}$ . The symmetric Laplacian matrix can be factored as $L=U\Lambda U^{T},$ where $U=(u_{0}, \ldots,u_{N-1})$ is the eigenvectors, also called graph Fourier modes, and $\Lambda=diag(\lambda_{0}, \ldots, \lambda_{N-1})$ is the corresponding diagonal matrix, indicating the frequency of the graph mode. The graph convolutional of the input signal $x$with a filter $g$ is defined as

$x*g=U \left( U^{T}x⨀U^{T}g \right)$ (2)

where $⨀$ denotes the Hadamard product. The graph convolutional can be simplified as $x*g_{\theta}=Ug_{\theta}U^{T}g$, where $g_{\theta}=diag\left( U^{T}g \right)$.

In order to reduce the computation complexity and avoids the graph Fourier basis computation, ChebNet defines the filter as Chebyshev polynomials of the diagonal matrix of eigenvalues ^6^. The graph convolutional with the Chebyshev polynomials filter is

$x*g_{\theta}=U\sum_{i=1}^{K} \theta_{i}T_{k}(\tilde{\Lambda}))U^{T}x=\sum_{i=1}^{K} \theta_{i}T_{i}(\tilde{L})x$ (3)

where $\tilde{\Lambda}=\frac{2\Lambda}{\lambda_{max}}-I$, $\tilde{L}=\frac{2L}{\lambda_{max}}-I$, $\lambda_{max}$ is the largest eigenvalue and $\theta_{i}$ is the learnable parameter.

Kipf et al. introduced a first-order approximation of ChebNet, assuming $K=1$ and $\lambda_{max}=2$ ^7^. The equation is simplified as

$x*g_{\theta}=\theta_{0}x-\theta_{1}D^{-\frac{1}{2}}AD^{-\frac{1}{2}}x$ (4)

1st ChebNet assumes $\theta=\theta_{0}=-\theta_{1}$, to avoid overfitting. Under this definition, the graph convolutional layer is

$X^{k+1}=\tilde{A}X^{k}\Theta^{k}$ (5)

where $\tilde{A}=I+D^{-\frac{1}{2}}AD^{-\frac{1}{2}}$.

**Definition of the population graph**

Our construction of the population graph is an extension of Parisot, S. et al^8^. GCN based on a population graph that combined imaging and non-imaging data is proven to be effective in disease classification ^9-11^. In these population graphs, each subject was regarded as a node and edges were constructed by phenotypic measures and subject similarity. The population adjacency matrix is defined as:

$A=sim\left( x_{u},x_{v} \right)\sum_{n} \mathcal{H}\left( P_{n}\left( u \right),P_{n}\left( v \right) \right)$ (6)

$sim\left( x_{u},x_{v} \right)=exp(-\frac{\left[ corr\left( x_{u},x_{v} \right) \right]^{2}}{2\sigma^{2}})$ (7)

$\mathcal{H}\left( P_{n}\left( u \right),P_{n}\left( v \right) \right)=\left\{ \begin{aligned} 1 if P_{n}\left( u \right)=P_{n}\left( v \right) \\ 0 otherwise. \end{aligned} \right.$ (8)

where $sim(\cdot)$ is the similarity function between features, $\sigma$ determines the width of the kernel. We used this similarity function to measure the correspondence of the FNC feature between every two subjects. $\mathcal{H}$represents the measured distance between non-image information. The definition of $\mathcal{H}$indicates that the subjects in the same group(male) tend to have more similar attributes, such as brain connectivity patterns, than subjects in a different group(female). And the edges’ weights were assigned with the same impact coefficient if pairwise subjects have the phenotypic information. These non-imaging measures can provide essential information to determine a neighborhood system that explains the similarity between the subjects. Here, we followed the definition of Parisot and assigned nodes’ features with FNC. To model the individual connectivity, we selected two phenotypic measures $P_{n}$: gender and age to define the adjacency matrix.

**Deep K-Means**

Deep K-means is the key clustering part of the GCN-BSD. Note that we didn’t use the association between individuals such as gender or age to cluster, instead, the clustering is based on the FNC features learned by GCN-BSD. Assume $K$ is the number of clusters, $r_{k}$represents the cluster $k,$ and $\mathcal{R=}\left\{ r_{1},\ldots, r_{k} \right\}$. For any $h{, c}_{f}\left( h\mathcal{,R} \right)$ gives the closest cluster of $h$ according to $f.$ The clustering loss corresponding to the K-Means objective function for embeddings can be denoted as $f\left( h,c_{f}\left( h;\mathcal{R} \right) \right)=\sum_{k} f_{k}\left( h; \mathcal{R} \right)$, where $f_{k}\left( h;\mathcal{R} \right)=f_{k}\left( h; r_{k} \right)$, if $r_{k}=c_{f}\left( h,\mathcal{R} \right)$, otherwise, $f_{k}\left( h\mathcal{;R} \right)=0$. However, this equation is not differentiable, unable to do end-to-end training. SoftMax function has been applied in several contexts as a differentiable surrogate ^12,13^. Here, we adopted the SoftMax function as follows:

$G_{k,f}\left( h,\alpha\mathcal{;R} \right)=\frac{e^{-\alpha f_{k}\left( h; r_{k} \right)}}{\sum_{k^{'}} e^{-\alpha f_{k}\left( h; r_{k^{'}} \right)}}$ (9)

$G_{k,f}$ is differentiable wrt $h\mathcal{, R}$ and continuous wrt $\alpha,$ with $\alpha\in(0,+\infty)$. Finally, the K-Means objective function can be denoted as follows:

$f\left( h,c_{f}\left( h;\mathcal{R} \right) \right)=\sum_{k} f_{k}\left( h; \mathcal{R} \right)G_{k,f}\left( h,\alpha\mathcal{;R} \right)$ (10)

Moreover, we have $\lim_{\alpha\to+\infty} f_{k}\left( h; \mathcal{R} \right)G_{k,f}\left( h,\alpha\mathcal{;R} \right)=f_{k}\left( h; \mathcal{R} \right)$. Equation (7) is fully differentiable wrt both $h$ and $\mathcal{R}$.

**GCN-BSD validation**

We tested the reliability of two clusters/biotypes within ABCD data by performing 10 times of 10-fold cross-validation. During each iteration, the data were partitioned into ten folds. Each fold was successively used as a test set while the remaining folds were used to do the clustering. After clustering the training set, we calculated the mean FNC of each group as their patterns and measured the Euclidean distance between each FNC in test set and two patterns, then divided this subject to its nearest group. In each realization, we used majority voting to ultimately determine which subgroup each subject belonged to. We evaluated the 10-fold clustering results through the consistency of mean FNC and individual divided result.

We tested the contribution of the population graph itself by using random data as the nodes’ feature instead of using FNC. We use two methods to configure random data: (1) Random data satisfy a normal distribution with 0 as the mean value and 1 as the standard deviation. (2) Random data satisfy a normal distribution with the same mean value and same standard deviation as the ADHD group and HC group, separately. Besides, we tested the contribution of the imaging data itself by using random data as the edges instead of using age and gender. We use two methods to configure random data: (1) Random edges satisfied the distribution of the real edges. (2) Fully connected graph as each node is connected to all the other nodes.

**ABCD biotypes validation**

Considering that there may be subjects who have taken medication and that subjects may have received influence from each other because of siblings or twin’s relationships. We analyzed the stability of the ABCD biotypes after excluding family relatedness and treatment factors.

To further substantiate our findings, we implemented additional restrictions based on the screening criteria referenced in the Cordova’s previous work ^5^. Cordova et al. primarily considered the following four points: (1) ADHD-Tier 1: Met ADHD-current on the KSADS-COMP. (DSM-5 Criterion A). Exclude ADHD-past-only. This was hypothesized to yield a prevalence similar to other parent-report-only studies (e.g. about 8–9%). (2) ADHD-Tier 2: ADHD-1 + rule out unspecified schizophrenia spectrum and other psychotic disorder (hereafter, “psychosis”), bipolar disorder, or estimated IQ<70 (DSM-5 Criterion E). This was not expected to much change the prevalence estimate. (3) ADHD-Tier-3: ADHD-2 + teacher BPM T-score ≥ 65 (DSM-5 Criterion C). (4) ADHD-Tier 4: ADHD-3 + parent CBCL attention scale or ADHD DSM5 scale T ≥ 65 (DSM-5 Criterion E). Here we expected prevalence to drop to that predicted by the more stringent studies cited earlier (3–4%).

**Different medication sensitivity between two biotypes**

We calculated the reduction rate for RS-IV and CPRS from week1 to week 8, with baseline scales being regressed out. Reduction rate is calculated as {baseline - post treatment)/baseline. Specifically, when baseline scale is 0 and current scale is 0, we recorded the reduction rate as nan. While when baseline scale is 0 and current scale is larger than 0, we recorded the reduction rate as -1.

**Results**

**The validation of GCN-BSD**

As shown in **Fig. S3**, the results of 10-fold cross-validation showed that the mean FNC patterns show extremely high correlation between all ABCD dataset and 10-fold subsets (r=0.9999, p<10^-300^ for biotype 1, and r=0.9992, p<10^-300^ for biotype 2). As for the mean FNC difference between biotypes and HC, the correlation is slightly lower, but still objectively high (r=0.9898, p<10^-300^ for biotype 1, and r=0.9877, p<10^-300^ for biotype 2). And in each realization, 93% subjects were divided into the same subgroup as the results from all data on average.

**
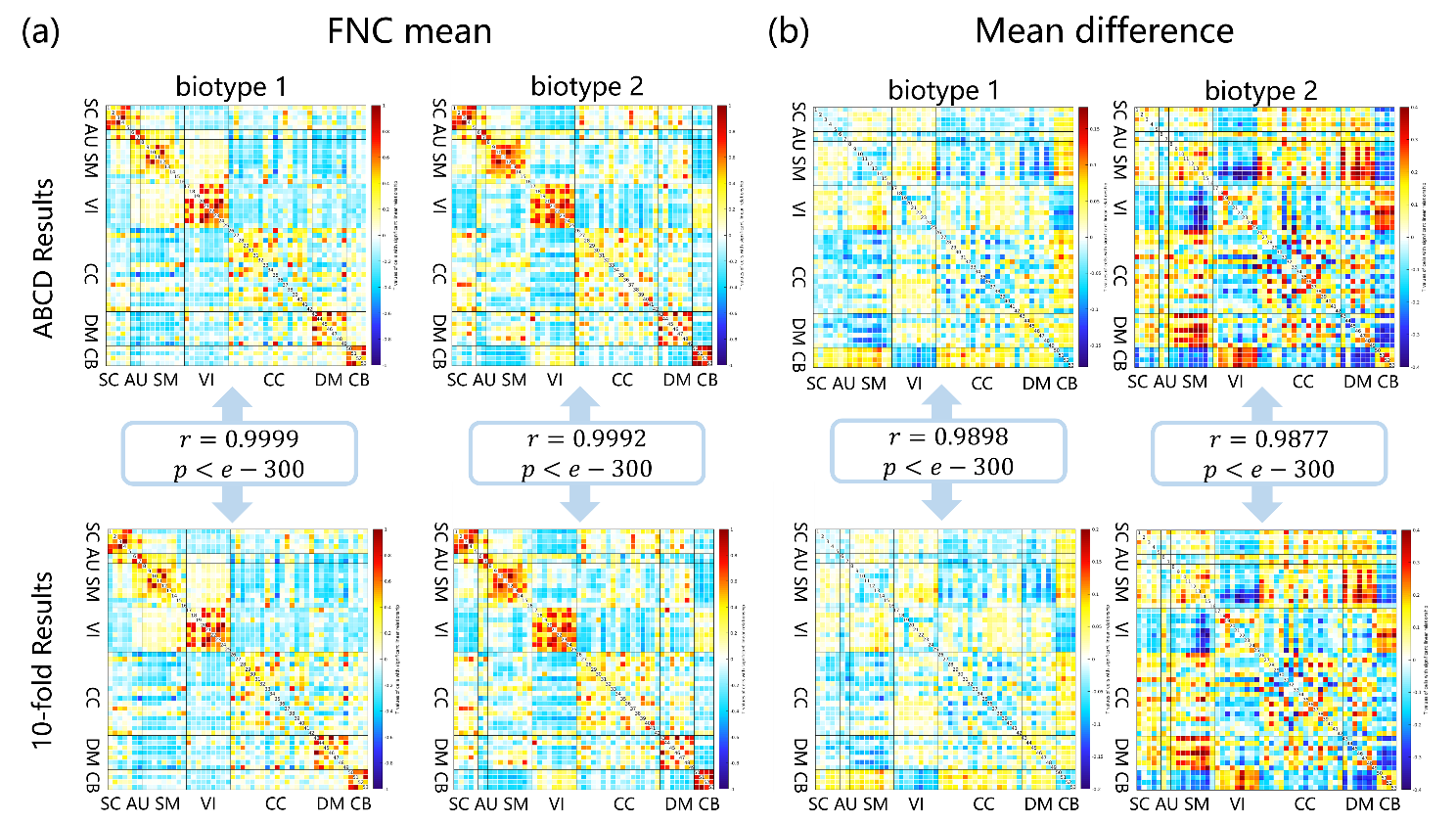
**

**Figure S3. (a)** The mean FNC of 2 biotypes identified from all data of ABCD and 10-fold data. **(b)** The mean differences of FNC between 2 biotypes and HC identified from all data of ABCD and 10-fold data.

As shown in Fig. S4 and S5, the results of testing the contribution of population graph itself or FNC imaging with only random data showed that clustering cannot be performed, and meaningful results cannot be obtained. Regardless of whether we use random numbers that conform to the standard normal distribution or to the normal distribution as the two sets of data, the features learned from the embedding layer of GCN-BSD exhibit a disorganization and contain no valuable information. Even, when we use a fully connected graph, the model is no longer able to classify ADHD into two biotypes but prefers to classify all ADHD samples into one biotype. In the case of fully connected graph, this model might amplify the difference between HC and ADHD. Specifically, the embedding feature of biotype 1 and biotype 2 is intermixed and indistinguishable, while in Fig.2e, the two distributions of data exhibit completely different forms and are distinguishable.

**
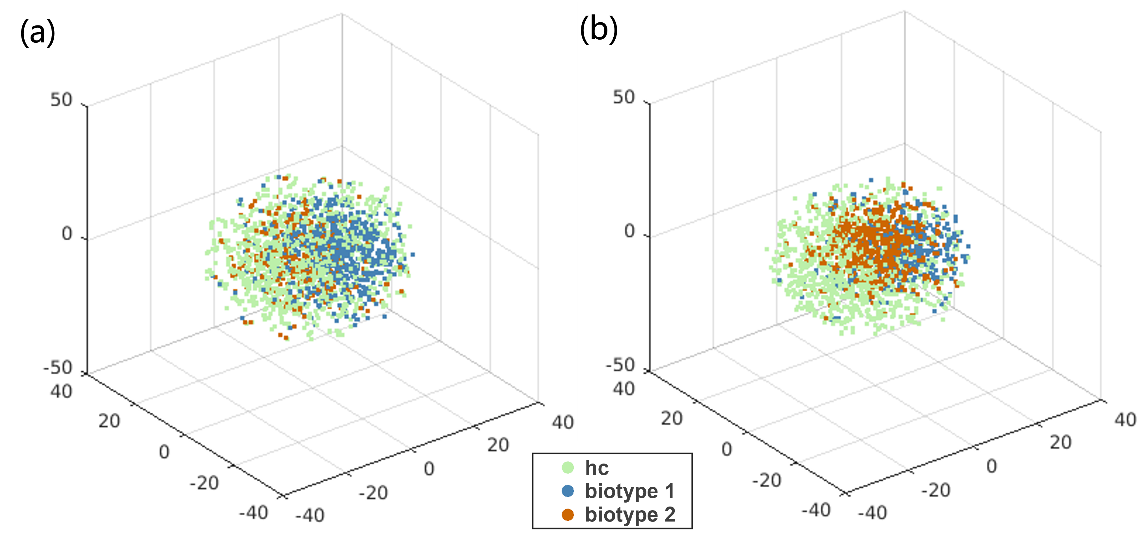
**

**Figure S4.** Clustering results of the embedding features learned from GCN-BSD for ADHD biotypes and HC visualized by t-SNE. **(a)** Random data satisfy a normal distribution with 0 as the mean value and 1 as the standard deviation. **(b)** Random data satisfy a normal distribution with the same mean value and same standard deviation as the ADHD group and HC group, separately.


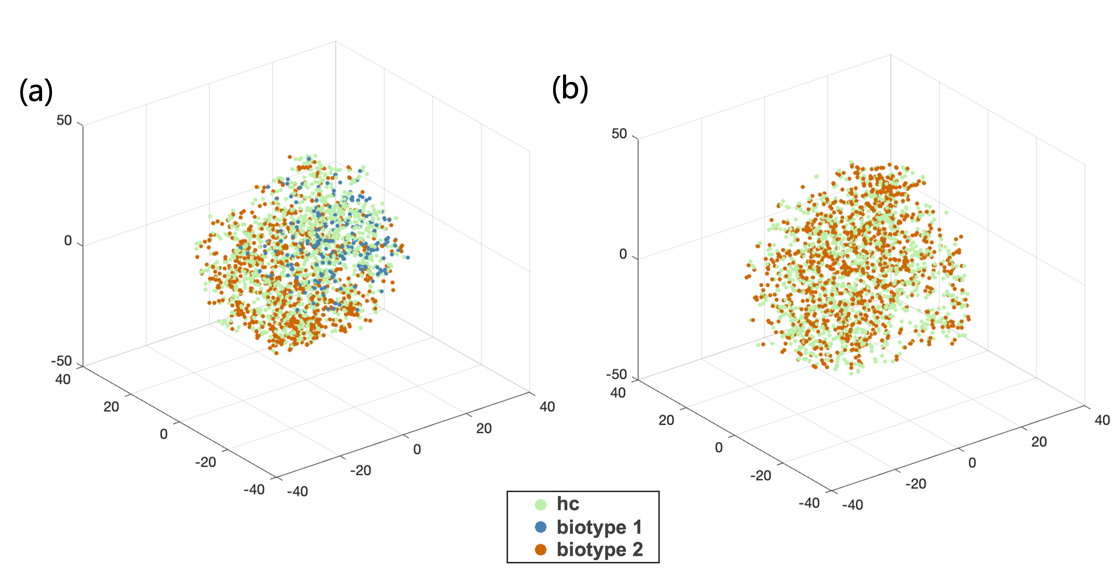


**Figure S5.** Clustering results of the embedding features learned from GCN-BSD for ADHD biotypes and HC visualized by t-SNE. (a) Random edges satisfied the distribution of the real edges. (b) Fully connected graph as each node is connected to all the other nodes.

**The validation of ABCD biotypes**

Based on the results of Cordova’s enhanced screening process, we analyzed the outcomes for tier 1, tier 2, tier 3 and tier 4:


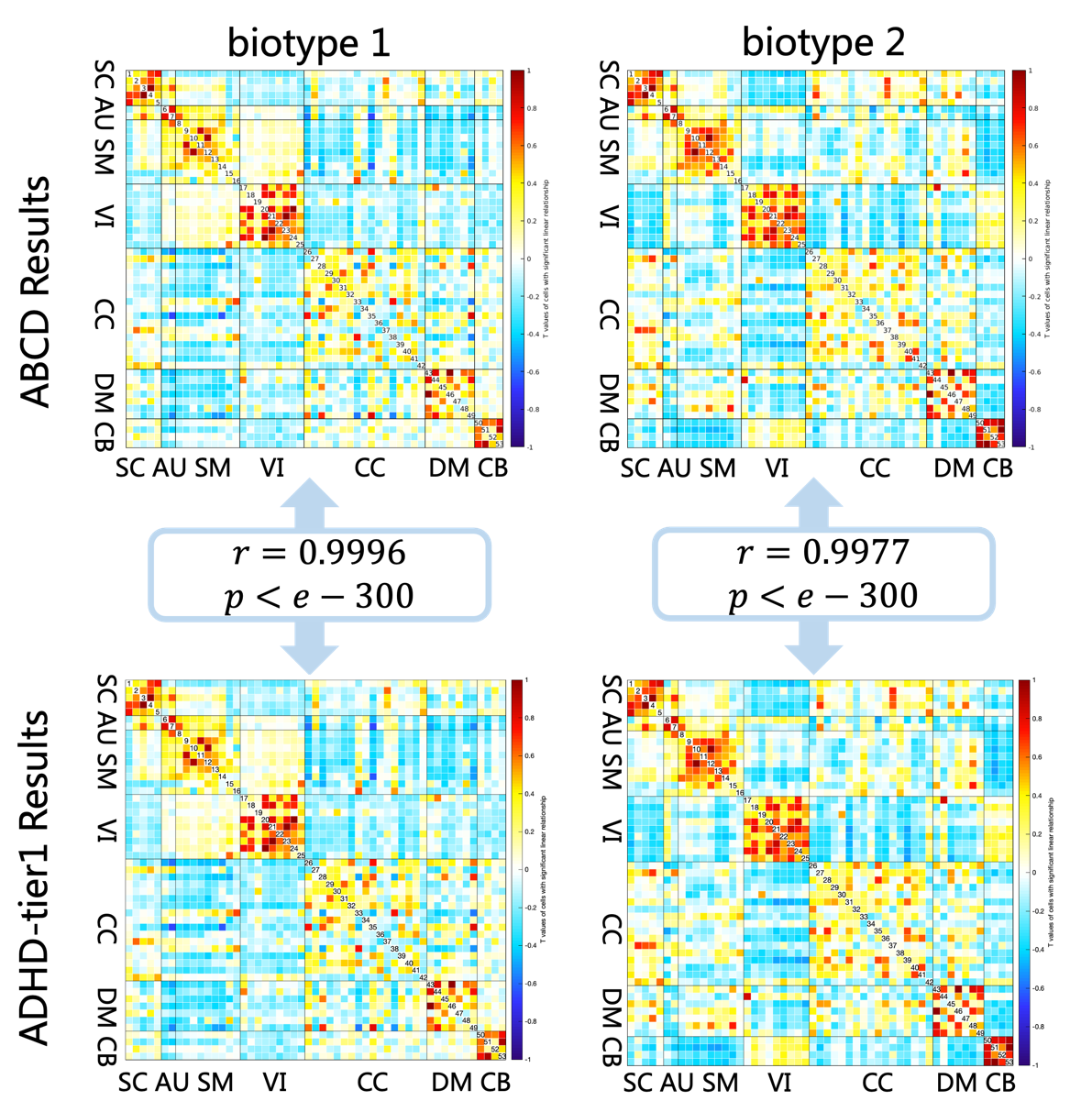


**Figure S6.** The original biotype FNC patterns showed high correlation with the ADHD-tier 1 biotype FNC patterns (r=0.9996, p<10^-300^ for biotype 1, and r=0.9977, p<10^-300^ for biotype 2).

**ADHD-Tier 1:**

After applying more stringent criteria for the diagnosis of ADHD, we found that 380 participants remained in biotype 1 and 122 participants remained in biotype 2. We analyzed the similarity between the original biotype FNC patterns and the ADHD-tier 1 biotype FNC patterns and found high correlation in both biotype 1 and biotype 2 (r=0.9996, p<10^-300^ for biotype 1, and r=0.9977, p<10^-300^ for biotype 2), as shown in Fig. S6.


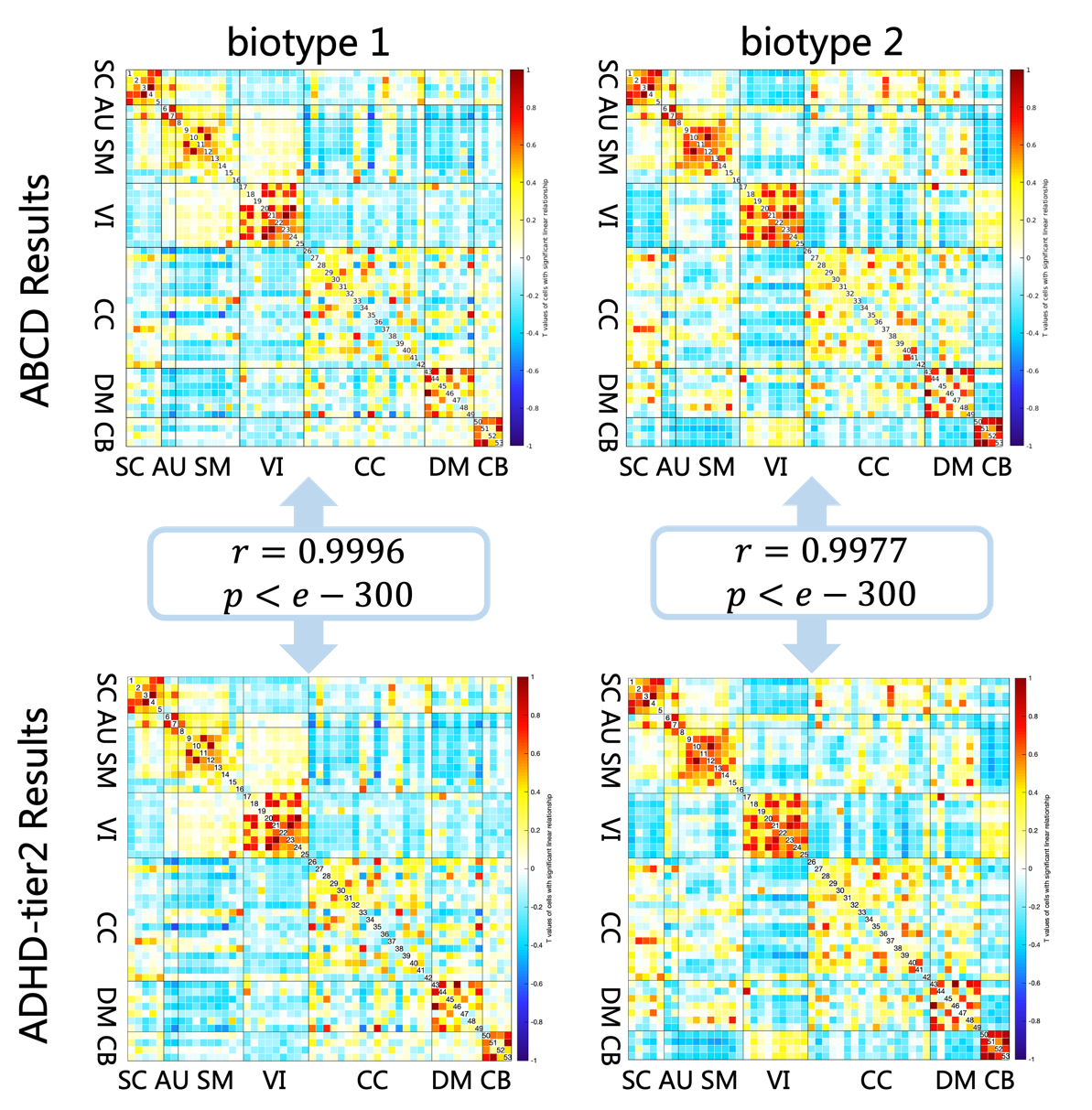


**Figure S7.** The original biotype FNC patterns showed high correlation with the ADHD-tier 2 biotype FNC patterns (r=0.9996, p<10^-300^ for biotype 1, and r=0.9977, p<10^-300^ for biotype 2).

**ADHD-Tier 2:**

After excluding patients with unspecified schizophrenia spectrum and other psychotic disorder, bipolar disorder, or estimated IQ<70, we found that 361 participants remained in biotype 1 and 115 participants remained in biotype 2. We analyzed the similarity between the original biotype FNC patterns and the ADHD-tier 2 biotype FNC patterns and also found high correlation in both biotype 1 and biotype 2 (r=0.9996, p<10^-300^ for biotype 1, and r=0.9977, p<10^-300^ for biotype 2), as shown in Fig. S7.


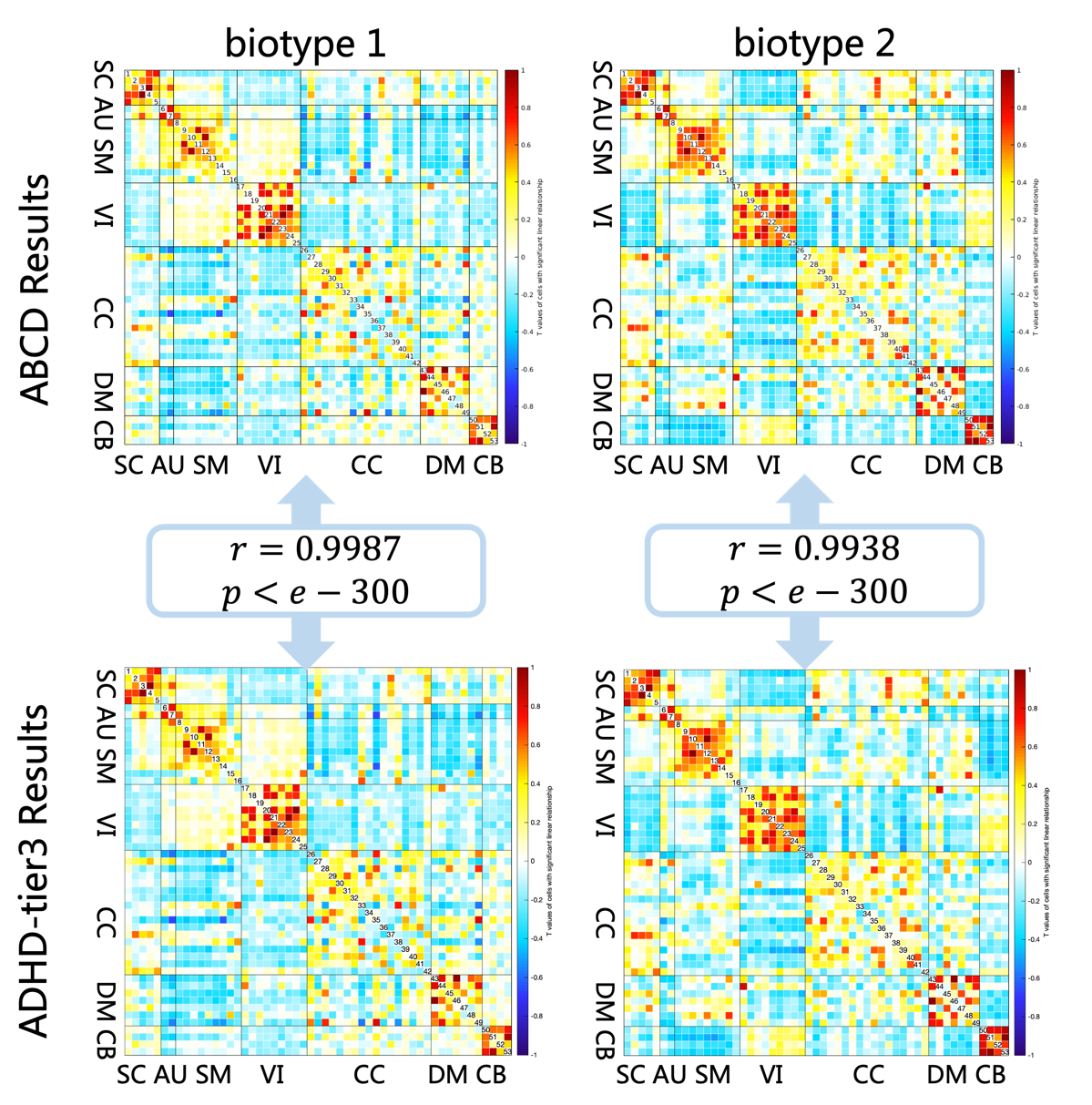


**Figure S8.** The original biotype FNC patterns showed high correlation with the ADHD-tier 3 biotype FNC patterns (r=0.9987, p<10^-300^ for biotype 1, and r=0.9938, p<10^-300^ for biotype 2).

**ADHD-Tier 3:**

Additionally, the screening requirements for participants in tier 3 included teacher BPM T-score ≥ 65. We found that 360 patients met our imaging data quality criteria, and overlapped with ADHD-tier 2. 165 participants remained in biotype 1 and 49 participants remained in biotype 2. And the correlation in both biotype 1 and biotype 2 is still high (r=0.9987, p<10^-300^ for biotype 1, and r=0.9938, p<10^-300^ for biotype 2).


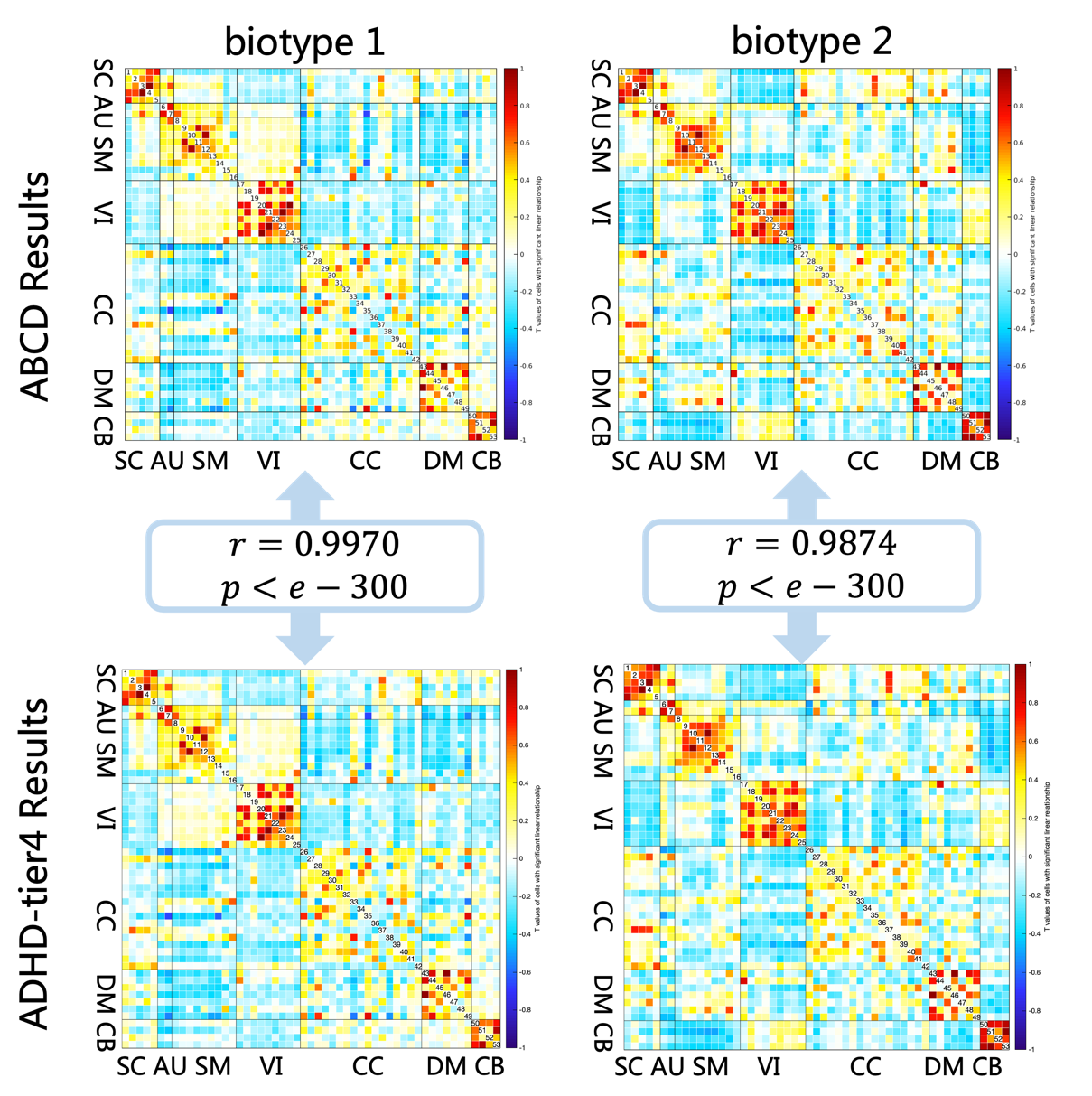


**Figure S9.** The original biotype FNC patterns showed high correlation with the ADHD-tier 4 biotype FNC patterns (r=0.9970, p<10^-300^ for biotype 1, and r=0.9874, p<10^-300^ for biotype 2).

**ADHD-Tier 4:**

Finally, the screening requirements for participants in tier 4 included t parent CBCL attention scale or ADHD DSM5 scale T ≥ 65. We found that 115 patients met our imaging data quality criteria and overlapped with ADHD-tier 4. 85 participants remained in biotype 1 and 30 participants remained in biotype 2. And the correlation in both biotype 1 and biotype 2 is r=0.9970, p<10^-300^for biotype 1, and r=0.9874, p<10^-300^ for biotype 2.


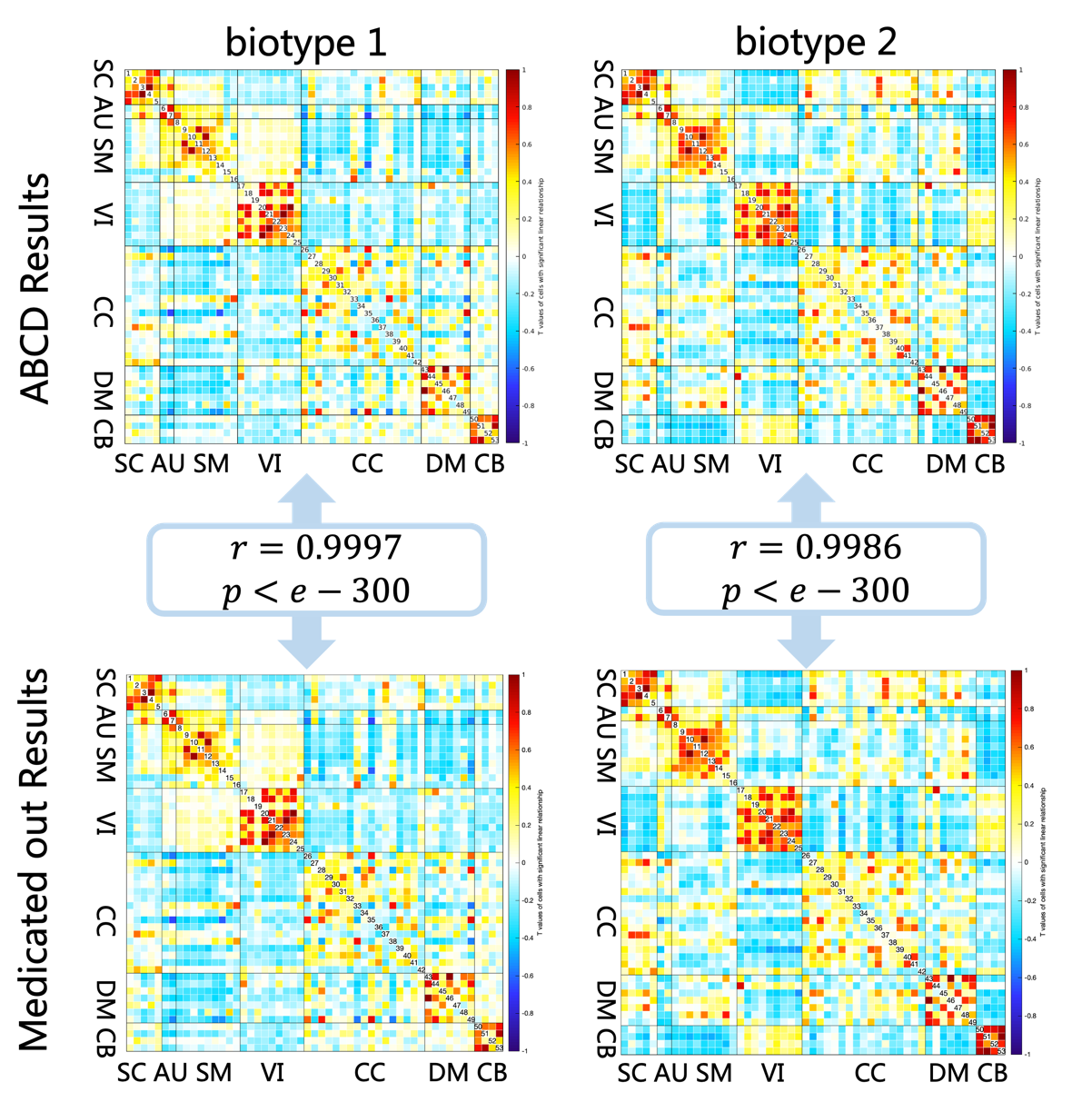


**Figure S10.** The original biotype FNC patterns showed high correlation with the FNC patterns excluding medicated participants.

After excluding the medicated participants, we retained 469 participants in the original biotype 1 and 161 participants in biotype 2. We performed a correlation analysis between the new results and the original results and found that our biotypes demonstrated a certain level of stability with r=0.9997, p<10^-300^ for biotype 1, and r=0.9986, p<10^-300^ for biotype 2 (Fig. S10).


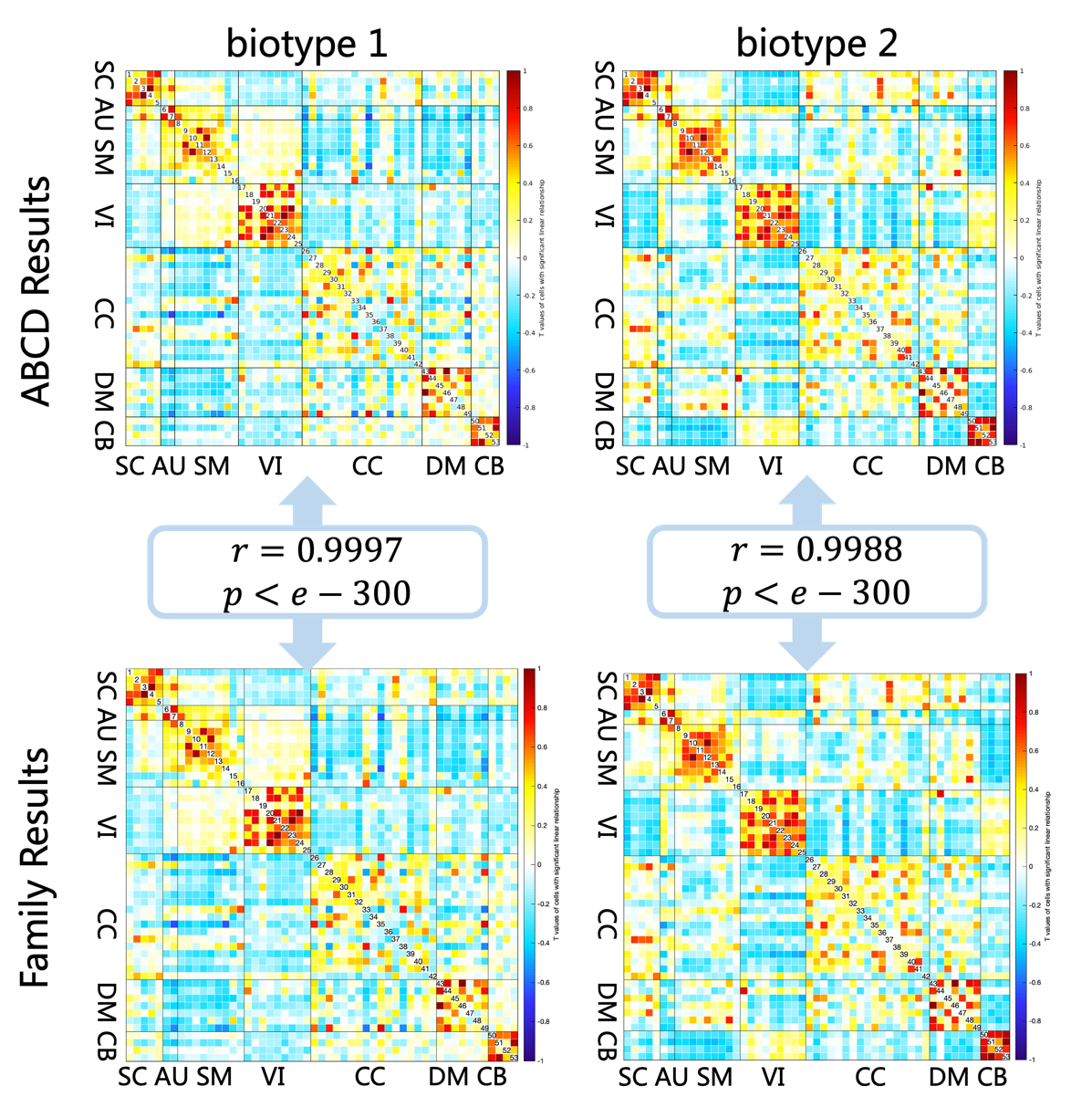


**Figure S11.** The original biotype FNC patterns showed high correlation with the FNC patterns when considering family relatedness.

After choosing one child per family, we retained 411 participants in the original biotype 1 and 132 participants in biotype 2. We performed a correlation analysis between the new results and the original results and found that our biotypes also demonstrated a certain level of stability with r=0.9997, p<10^-300^ for biotype 1, and r=0.9988, p<10^-300^ for biotype 2, as shown in Fig. S11.

**The correlation between consistent discriminative FNC and cognition and symptom scales.**

The consistent discriminative FNCs between biotype 1 and biotype 2 were highly corrected with several cognition scales, as shown in **Table S10**.

**Table S10. The correlation between FNC and scales.**

| **CPRS-ADHD index** | $r=0.20$  $p=0.03$ |  |  | $r=0.20$  $p=0.032$ |
| --- | --- | --- | --- | --- |
| **CPRS-impulsivity–hyperactivity** | $r = 0.22$  $p=0.02$ |  |  | $r=0.34$  $p=0.0002$ |
| **RS-inattentive** |  |  |  | $r=0.40$  $p=3.00e-5$ |
| **RS-hyperactive/impulsive** | $r=0.20$  $p=0.03$ |  |  | $r= 0.29$  $p=0.0008$ |
| **PVT** | $r = -0.15$  $p=6.38e-7$ | $r = -0.13$  $p=2.95e-7$ | $r = -0.13$  $p=4.61e-6$ | $r=-0.14$  $p=4.62e-6$ |
| **PCPST** | $r=-0.12$  $p=0.0002$ | $r = -0.11$  $p = 0.0003$ |  |  |
| **LSWMT** | $r=-0.10$  $P=0.0008$ | $r=-0.10$  $p = 0.0009$ |  | $r=-0.13$  $P=0.0009$ |
| **DCCST** | $r=-0.11$  $p=0.0002$ | $r = -0.12$  $p=4.92e-5$ | $r=-0.14$  $p=0.0001$ | $r=-0.13$  $p=2.35e-5$ |
| **crystallized intelligence** | $r=-0.13$  $p=8.46e-6$ | $r = 0.13$  $p=3.23e-5$ | $r = 0.13$  $p=3.23e-5$ | $r=-0.13$  $p=1.60e-5$ |
| **fluid intelligence** | $r=-0.14$  $p=3.44e-6$ | $r=-0.13$  $p=1.65e-5$ | $r=0.14$  $p=1.18e-5$ | $r=-0.14$  $p=3.37e-6$ |
| **total intelligence** | $r = -0.16$  $p= 2.47e-7$ | $r=-0.16$  $p=3.78e-7$ | $r=0.13$  $p=2.41e-5$ | $r=-0.16$  $p=9.47e-8$ |
|  | superior parietal lobule – precuneus | superior parietal lobule and postPapaerior cingulate cortex | middle temporal gyrus and paracentral lobule | cerebellum-fusiform gyrus |

**The group difference in socio-economic status.**


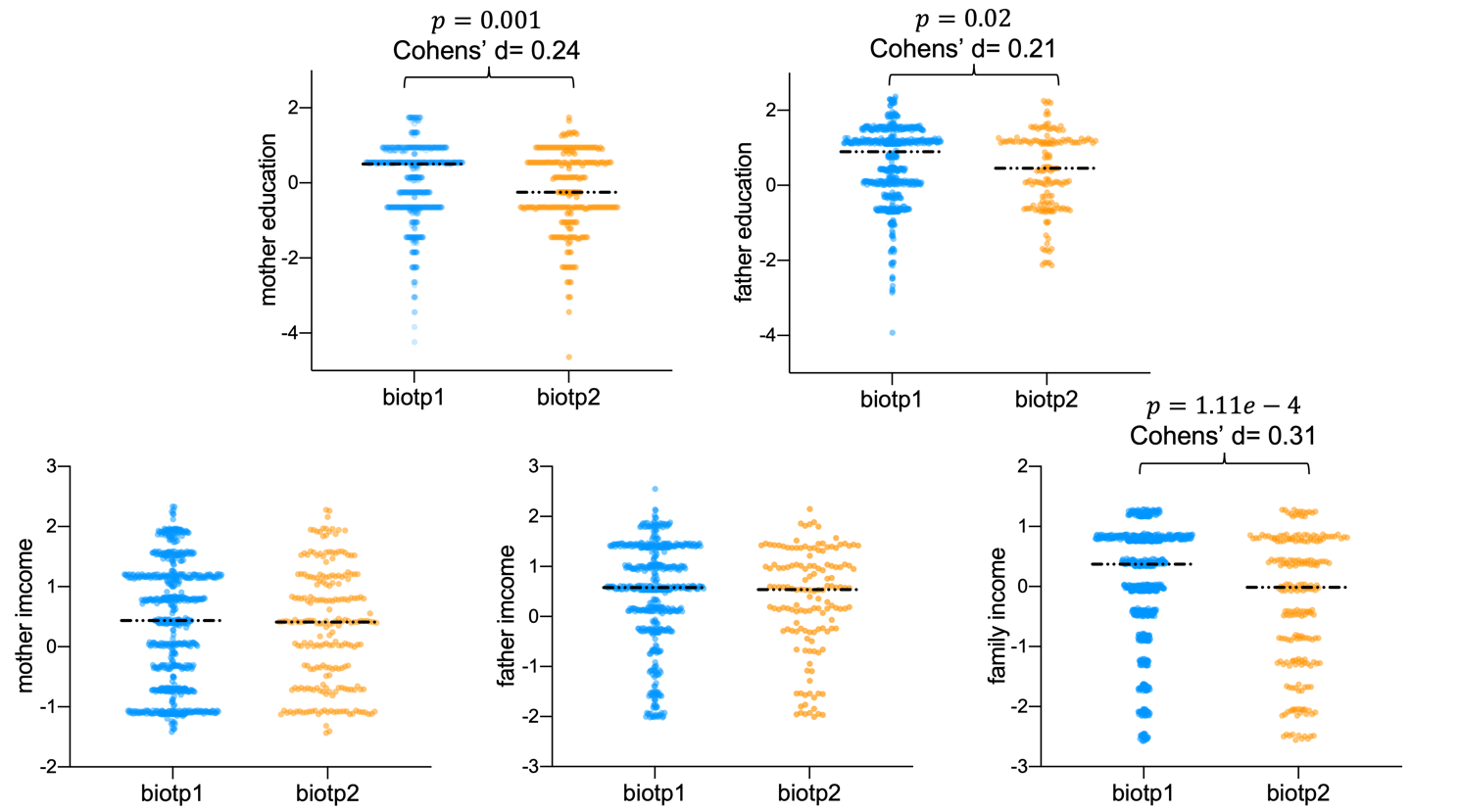


**Figure S12.** Significant group differences between biotype 1 and biotype 2 considering family exposures including mother education, father education and family income.

**Table S11. socio-economic status of the biotypes from ABCD.**

| **Characteristics** | **biotype 1** | **biotype 2** | **P-value** | **Cohen’s d** |
| --- | --- | --- | --- | --- |
| mo_education | 16.76±2.45 | 16.14±2.64 | 0.001 | 0.24 |
| fa_education | 16.38±2.72 | 15.80±2.88 | 0.02 | 0.21 |
| family_income | 6.46±2.65 | 7.23±2.31 | 1.11×10^-4^ | 0.31 |

**The results of biotypes under medication**

**Table S12. Demographic information and symptom scale of the biotypes from PKU.**

| **Variables** |  | **Biotype1 (n=26)** | | **Biotype2(n=18)** | **P-value** |
| --- | --- | --- | --- | --- | --- |
| **Demographics** | Age(years) | | 11.19±2.29 | 11.22±2.10 | 0.96 |
|  | Gender(M/F) | | 17/9 | 15/3 | 0.18 |
| **Baseline** | RS-inattention | | 28.03±3.27 | 27.27±2.37 | 0.38 |
|  | RS-IH | | 20.27±6.26 | 20.27±5.11 | 0.99 |
|  | RS-total | | 48.31±8.68 | 47.76±6.40 | 0.74 |
|  | CPRS-Conduct Problems | | 12.38±6.09 | 13.26±5.81 | 0.63 |
|  | CPRS-Learning Problems | | 7.34±2.50 | 7.18±1.98 | 0.81 |
|  | CPRS-Psychosomatic Problems | | 2.30±2.02 | 1.44±1.69 | 0.13 |
|  | CPRS-Impulsivity–Hyperactivity | | 5.42±3.04 | 6.89±2.99 | 0.12 |
|  | CPRS-Anxiety | | 2.31±1.72 | 2.01±1.88 | 0.60 |
|  | CPRS-ADHD index | | 14.19±5.19 | 15.09±4.24 | 0.53 |
| **Week 1** | RS-inattention | | 22.66±5.70 | 22.80±4.53 | 0.93 |
|  | RS-IH | | 16.33±4.80 | 17.18±5.81 | 0.61 |
|  | RS-total | | 38.99±9.47 | 39.99±9.31 | 0.73 |
|  | CPRS-Conduct Problems | | 10.27±4.65 | 11.94±6.87 | 0.38 |
|  | CPRS-Learning Problems | | 6.36±2.24 | 6.49±2.25 | 0.85 |
|  | CPRS-Psychosomatic Problems | | 1.59±1.94 | 0.98±1.16 | 0.20 |
|  | CPRS-Impulsivity–Hyperactivity | | 3.77±2.88 | 4.57±2.77 | 0.36 |
|  | CPRS-Anxiety | | 1.77±1.53 | 2.16±2.01 | 0.49 |
|  | CPRS-ADHD index | | 10.53±4.51 | 11.38±4.97 | 0.57 |
| **Week 2** | RS-inattention | | 22.35±5.04 | 23.01±4.76 | 0.66 |
|  | RS-IH | | 16.27±4.70 | 16.44±5.35 | 0.91 |
|  | RS-total | | 38.62±8.89 | 39.45±8.75 | 0.76 |
|  | CPRS-Conduct Problems | | 10.02±4.36 | 11.88±6.40 | 0.29 |
|  | CPRS-Learning Problems | | 6.04±2.44 | 6.00±2.19 | 0.96 |
|  | CPRS-Psychosomatic Problems | | 1.66±1.80 | 1.53±1.82 | 0.82 |
|  | CPRS-Impulsivity–Hyperactivity | | 3.89±2.50 | 4.39±2.70 | 0.54 |
|  | CPRS-Anxiety | | 1.76±1.45 | 2.11±1.53 | 0.46 |
|  | CPRS-ADHD index | | 10.06±4.53 | 10.63±4.45 | 0.68 |
| **Week 3** | RS-inattention | | 20.88±5.21 | 22.05±4.85 | 0.45 |
|  | RS-IH | | 16.19±5.45 | 16.27±4.31 | 0.95 |
|  | RS-total | | 37.08±9.87 | 38.32±7.42 | 0.64 |
|  | CPRS-Conduct Problems | | 9.15±5.05 | 10.77±6.12 | 0.36 |
|  | CPRS-Learning Problems | | 5.53±2.75 | 5.98±2.11 | 0.55 |
|  | CPRS-Psychosomatic Problems | | 1.62±1.86 | 0.91±1.23 | 0.14 |
|  | CPRS-Impulsivity–Hyperactivity | | 3.58±2.62 | 4.32±1.72 | 0.26 |
|  | CPRS-Anxiety | | 1.46±1.70 | 1.75±1.59 | 0.56 |
|  | CPRS-ADHD index | | 9.19±4.86 | 10.43±3.43 | 0.33 |
| **Week 4** | RS-inattention | | 19.96±6.29 | 21.89±4.46 | 0.24 |
|  | RS-IH | | 15.27±5.82 | 16.78±4.75 | 0.34 |
|  | RS-total | | 35.24±11.60 | 38.67±7.53 | 0.24 |
|  | CPRS-Conduct Problems | | 9.35±5.06 | 11.33±6.61 | 0.29 |
|  | CPRS-Learning Problems | | 5.53±2.97 | 5.94±1.92 | 0.58 |
|  | CPRS-Psychosomatic Problems | | 1.73±1.82 | 1.28±1.81 | 0.42 |
|  | CPRS-Impulsivity–Hyperactivity | | 3.34±2.78 | 4.50±1.95 | 0.11 |
|  | CPRS-Anxiety | | 1.81±1.47 | 2.33±1.81 | 0.31 |
|  | CPRS-ADHD index | | 9.31±4.64 | 11.11±3.32 | 0.14 |
| **Week 8** | RS-inattention | | 19.07±6.70 | 21.06±6.73 | 0.36 |
|  | RS-IH | | 14.18±5.62 | 17.00±6.29 | 0.14 |
|  | RS-total | | 33.25±11.68 | 38.32±12.12 | 0.21 |
|  | CPRS-Conduct Problems | | 9.00±6.11 | 11.56±6.71 | 0.21 |
|  | CPRS-Learning Problems | | 4.65±2.94 | 6.00±2.03 | 0.08 |
|  | CPRS-Psychosomatic Problems | | 1.11±1.51 | 0.89±1.64 | 0.64 |
|  | CPRS-Impulsivity–Hyperactivity | | 2.96±2.16 | 4.66±2.05 | 0.01 |
|  | CPRS-Anxiety | | 1.62±1.63 | 1.67±1.57 | 0.91 |
|  | CPRS-ADHD index | | 8.38±5.06 | 10.83±3.70 | 0.07 |

**Table S13.** **Raw scale reduction rate of the bio-subjects under medication from PKU.**

| **Variables** |  | **Biotype1 (n=26)** | | **Biotype2(n=18)** | **P-value** |
| --- | --- | --- | --- | --- | --- |
| **Week 1** | RS-inattention | | 0.19±0.18 | 0.16±0.16 | 0.60 |
|  | RS-IH | | 0.16±0.22 | 0.13±0.24 | 0.69 |
|  | RS-total | | 0.19±0.16 | 0.16±0.16 | 0.57 |
|  | CPRS-Conduct Problems | | 0.16±0.18 | 0.14±0.20 | 0.62 |
|  | CPRS-Learning Problems | | 0.16±0.18 | 0.14±0.20 | 0.81 |
|  | CPRS-Psychosomatic Problems | | 0.16±0.17 | 0.14±0.21 | 0.48 |
|  | CPRS-Impulsivity–Hyperactivity | | 0.16±0.17 | 0.14±0.21 | 0.83 |
|  | CPRS-Anxiety | | 0.15±0.17 | 0.16±0.20 | 0.89 |
|  | CPRS-ADHD index | | 0.15±0.18 | 0.14±0.21 | 0.85 |
| **Week 2** | RS-inattention | | 0.20±0.17 | 0.16±0.18 | 0.38 |
|  | RS-IH | | 0.16±0.22 | 0.15±0.18 | 0.90 |
|  | RS-total | | 0.19±0.16 | 0.17±0.23 | 0.58 |
|  | CPRS-Conduct Problems | | 0.17±0.17 | 0.17±0.19 | 0.70 |
|  | CPRS-Learning Problems | | 0.17±0.17 | 0.17±0.19 | 0.79 |
|  | CPRS-Psychosomatic Problems | | 0.18±0.17 | 0.12±0.18 | 0.29 |
|  | CPRS-Impulsivity–Hyperactivity | | 0.17±0.17 | 0.17±0.19 | 0.15 |
|  | CPRS-Anxiety | | 0.17±0.17 | 0.19±0.18 | 0.59 |
|  | CPRS-ADHD index | | 0.17±0.17 | 0.17±0.19 | 0.97 |
| **Week 3** | RS-inattention | | 0.25±0.19 | 0.19±0.18 | 0.29 |
|  | RS-IH | | 0.17±0.26 | 0.17±0.20 | 0.94 |
|  | RS-total | | 0.22±0.19 | 0.19±0.16 | 0.49 |
|  | CPRS-Conduct Problems | | 0.21±0.17 | 0.19±0.19 | 0.47 |
|  | CPRS-Learning Problems | | 0.21±0.17 | 0.19±0.19 | 0.37 |
|  | CPRS-Psychosomatic Problems | | 0.22±0.17 | 0.18±0.23 | 0.66 |
|  | CPRS-Impulsivity–Hyperactivity | | 0.19±0.17 | 0.19±0.19 | 0.59 |
|  | CPRS-Anxiety | | 0.20±0.17 | 0.21±0.20 | 0.51 |
|  | CPRS-ADHD index | | 0.21±0.17 | 0.19±0.19 | 0.50 |
| **Week 4** | RS-inattention | | 0.28±0.23 | 0.20±0.16 | 0.13 |
|  | RS-IH | | 0.23±0.30 | 0.15±0.21 | 0.34 |
|  | RS-total | | 0.27±0.23 | 0.18±0.15 | 0.14 |
|  | CPRS-Conduct Problems | | 0.20±0.17 | 0.19±0.19 | 0.45 |
|  | CPRS-Learning Problems | | 0.20±0.17 | 0.19±0.19 | 0.26 |
|  | CPRS-Psychosomatic Problems | | 0.21±0.18 | 0.17±0.23 | 0.97 |
|  | CPRS-Impulsivity–Hyperactivity | | 0.19±0.16 | 0.19±0.19 | 0.90 |
|  | CPRS-Anxiety | | 0.19±0.16 | 0.20±0.20 | 0.33 |
|  | CPRS-ADHD index | | 0.20±0.17 | 0.19±0.19 | 0.26 |
| **Week 8** | RS-inattention | | 0.35±0.29 | 0.18±0.14 | 0.01 |
|  | RS-IH | | 0.29±0$.34$ | 0.15±0.19 | 0.11 |
|  | RS-total | | 0.33±0.30 | 0.17±0.13 | 0.02 |
|  | CPRS-Conduct Problems | | 0.21±0.22 | 0.23±0.23 | 0.38 |
|  | CPRS-Learning Problems | | 0.21±0.22 | 0.22±0.23 | 0.04 |
|  | CPRS-Psychosomatic Problems | | 0.23±0.22 | 0.16±0.16 | 0.46 |
|  | CPRS-Impulsivity–Hyperactivity | | 0.20±0.22 | 0.23±0.23 | 0.30 |
|  | CPRS-Anxiety | | 0.20±0.22 | 0.24±0.24 | 0.80 |
|  | CPRS-ADHD index | | 0.21±0.22 | 0.23±0.23 | 0.09 |

**Table S14.** **Scale reduction rate of the bio-subjects under medication from PKU.**

| **Variables** |  | **Biotype1 (n=26)** | | **Biotype2(n=18)** | **P-value** |
| --- | --- | --- | --- | --- | --- |
| **Week 1** | RS-inattention | | 0.15±0.18 | 0.13±0.16 | 0.68 |
|  | RS-IH | | 0.09±0.19 | 0.06±0.22 | 0.65 |
|  | RS-total | | 0.14±0.16 | 0.12±0.16 | 0.61 |
|  | CPRS-Conduct Problems | | -0.07±0.63 | -0.28±1.03 | 0.46 |
|  | CPRS-Learning Problems | | -0.11±0.36 | -0.12±0.38 | 0.89 |
|  | CPRS-Psychosomatic Problems | | 0.05±0.74 | -0.08±0.61 | 0.59 |
|  | CPRS-Impulsivity–Hyperactivity | | 0.11±0.73 | -0.05±0.79 | 0.50 |
|  | CPRS-Anxiety | | -0.15±1.16 | -0.13±0.84 | 0.95 |
|  | CPRS-ADHD index | | 0.11±0.35 | 0.06±0.35 | 0.64 |
| **Week 2** | RS-inattention | | 0.13±0.16 | 0.09±0.17 | 0.49 |
|  | RS-IH | | 0.09±0.19 | 0.10±0.21 | 0.89 |
|  | RS-total | | 0.13±0.15 | 0.11±0.17 | 0.63 |
|  | CPRS-Conduct Problems | | -0.08±0.53 | -0.22±0.58 | 0.44 |
|  | CPRS-Learning Problems | | 0.02±0.32 | -0.04±0.39 | 0.85 |
|  | CPRS-Psychosomatic Problems | | -0.07±0.95 | -0.36±0.92 | 0.38 |
|  | CPRS-Impulsivity–Hyperactivity | | -0.14±1.03 | 0.07±0.52 | 0.37 |
|  | CPRS-Anxiety | | -0.15±0.99 | -0.27±0.75 | 0.66 |
|  | CPRS-ADHD index | | 0.20±0.38 | 0.18±0.31 | 0.84 |
| **Week 3** | RS-inattention | | 0.13±0.18 | 0.09±0.17 | 0.42 |
|  | RS-IH | | 0.10±0.24 | 0.11±0.17 | 0.94 |
|  | RS-total | | 0.16±0.19 | 0.12±0.15 | 0.53 |
|  | CPRS-Conduct Problems | | 0.16±0.50 | 0.03±0.40 | 0.34 |
|  | CPRS-Learning Problems | | 0.17±0.35 | 0.09±0.26 | 0.38 |
|  | CPRS-Psychosomatic Problems | | 0.01±0.75 | 0.19±0.57 | 0.45 |
|  | CPRS-Impulsivity–Hyperactivity | | 0.09±0.76 | 0.08±0.21 | 0.97 |
|  | CPRS-Anxiety | | 0.08±1.23 | -0.11±0.82 | 0.58 |
|  | CPRS-ADHD index | | 0.30±0.38 | 0.23±0.23 | 0.42 |
| **Week 4** | RS-inattention | | 0.20±0.22 | 0.13±0.15 | 0.18 |
|  | RS-IH | | 0.18±0.29 | 0.11±0.19 | 0.31 |
|  | RS-total | | 0.24±0.23 | 0.15±0.14 | 0.14 |
|  | CPRS-Conduct Problems | | 0.11±0.50 | -0.05±0.56 | 0.32 |
|  | CPRS-Learning Problems | | 0.20±0.38 | 0.08±0.29 | 0.26 |
|  | CPRS-Psychosomatic Problems | | -0.19±1.20 | 0.11±0.93 | 0.82 |
|  | CPRS-Impulsivity–Hyperactivity | | 0.15±0.74 | 0.03±0.43 | 0.48 |
|  | CPRS-Anxiety | | -0.11±0.97 | 0.43±1.09 | 0.34 |
|  | CPRS-ADHD index | | 0.29±0.37 | 0.17±0.26 | 0.20 |
| **Week 8** | RS-inattention | | 0.19±0.28 | 0.04±0.13 | 0.03 |
|  | RS-IH | | 0.25±0$.34$ | 0.12±0.17 | 0.10 |
|  | RS-total | | 0.29±0.30 | 0.13±0.12 | 0.03 |
|  | CPRS-Conduct Problems | | 0.10±0.68 | -0.12±0.55 | 0.24 |
|  | CPRS-Learning Problems | | 0.32±0.39 | 0.09±0.32 | 0.04 |
|  | CPRS-Psychosomatic Problems | | 0.20±0.70 | -0.006±0.93 | 0.54 |
|  | CPRS-Impulsivity–Hyperactivity | | 0.18±0.58 | -0.19±0.55 | 0.04 |
|  | CPRS-Anxiety | | 0.02±0.90 | 0.13±0.74 | 0.66 |
|  | CPRS-ADHD index | | 0.35±0.37 | 0.15±0.32 | 0.06 |

**LME results of PKU biotypes**

**Table S15. LME results of RS-IA**

|  | *Sum Sq* | *Mean Sq* | *F value* | *P value* |
| --- | --- | --- | --- | --- |
| week | 1.18e-3 | 1.18e-3 | 0.08 | 0.78 |
| biotype | 5e-6 | 5e-6 | 3e-4 | 0.99 |
| **week: biotype** | 0.10 | 0.10 | 6.57 | **0.01*** |

**Table S16. LME results of RS-IH**

|  | **Sum Sq** | **Mean Sq** | **F value** | **P value** |
| --- | --- | --- | --- | --- |
| **week** | 0.30 | 0.30 | 15.78 | **1.04e-4*** |
| biotype | 2.53e-3 | 2.53e-3 | 0.13 | 0.72 |
| **week: biotype** | 0.11 | 0.11 | 5.95 | **0.02*** |

**Table S17. LME results of RS-Total**

|  | **Sum Sq** | **Mean Sq** | **F value** | **P value** |
| --- | --- | --- | --- | --- |
| **week** | 0.22 | 0.22 | 15.34 | **1.29e-4*** |
| biotype | 3.0e-4 | 3.0e-4 | 0.02 | 0.89 |
| **week: biotype** | 0.12 | 0.12 | 8.42 | **4.19e-3*** |

**Table S18. LME results of CPRS-Hindex**

|  | **Sum Sq** | **Mean Sq** | **F value** | **P value** |
| --- | --- | --- | --- | --- |
| **week** | 0.39 | 0.39 | 10.42 | **1.49e-3*** |
| biotype | 5e-5 | 5e-5 | 1.3e-3 | 0.97 |
| **week: biotype** | 0.20 | 0.20 | 5.27 | **0.02*** |

**Table S19. Three-way LME results of CPRS-psycho problem**

|  | **Sum Sq** | **Mean Sq** | **F value** | **P value** |
| --- | --- | --- | --- | --- |
| medicine | 0.06 | 0.06 | 0.15 | 0.70 |
| biotype | 0.12 | 0.12 | 0.32 | 0.57 |
| week | 0.17 | 0.17 | 0.45 | 0.50 |
| **med: biotp** | 1.85 | 1.85 | 4.89 | **0.03*** |
| med: week | 0.04 | 0.04 | 0.10 | 0.75 |
| week: biotype | 0.09 | 0.09 | 0.23 | 0.63 |

**Table S20. Three-way LME results of CPRS-IH**

|  | Sum Sq | Mean Sq | F value | P value |
| --- | --- | --- | --- | --- |
| medcine | 0.67 | 0.67 | 4.87 | **0.03*** |
| biotype | 7.2e-4 | 7.2e-4 | 5.3e-3 | 0.94 |
| week | 0.06 | 0.06 | 0.41 | 0.52 |
| med:biotp | 0.03 | 0.03 | 0.02 | 0.88 |
| med:week | 7.06e-3 | 7.06e-3 | 0.05 | 0.82 |
| week:biotype | 3.04e-3 | 3.04e-3 | 2.82 | 0.10 |

**Table S21. LME results of CPRS-ADHD-index**

|  | Sum Sq | Mean Sq | F value | P value |
| --- | --- | --- | --- | --- |
| medcine | 0.18 | 0.18 | 4.96 | 0.03* |
| biotype | 9.5e-4 | 9.5e-4 | 0.03 | 0.87 |
| week | 0.39 | 0.39 | 10.74 | 1.35e-3* |
| med:biotp | 1.84e-3 | 1.84e-3 | 0.05 | 0.82 |
| med:week | 0.01 | 0.01 | 0.29 | 0.59 |
| week:biotype | 0.14 | 0.14 | 3.94 | 0.05 |

**Table S22. LME results of CPRS-ADHD-index+CPRS-IH**

|  | Sum Sq | Mean Sq | F value | P value |
| --- | --- | --- | --- | --- |
| medcine | 1.41 | 1.41 | 5.29 | 0.02* |
| biotype | 9.8e-4 | 9.8e-4 | 0.004 | 0.95 |
| week | 0.14 | 0.14 | 0.55 | 0.46 |
| med:biotp | 0.004 | 0.004 | 0.02 | 0.90 |
| med:week | 0.04 | 0.04 | 0.14 | 0.71 |
| week:biotype | 0.98 | 0.98 | 3.69 | 0.06 |


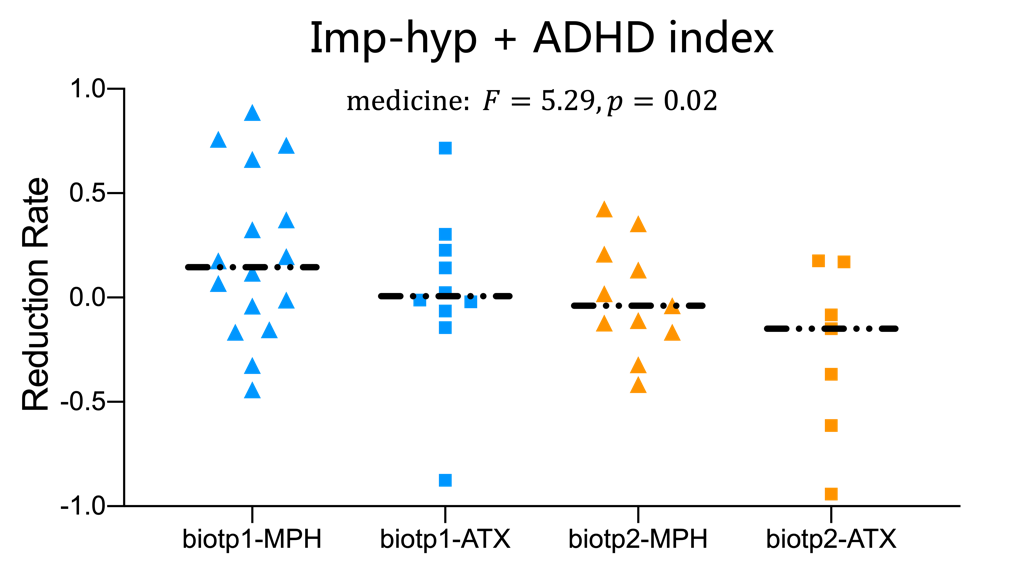


**Figure S13.** The reduction rate of using different medication in two biotypes when CPRS-impulsivity-hyperactivity and CRPS-ADHD index are averaged.

**The comparation of full three-way LME model and reduced LME model**

We identified full model as: score ~ week * sub *med + (1 | ID), and reduced model as: score ~ week * sub + med * sub + week*med + (1 | ID). The full model includes all interaction terms, and the reduced model excludes the three-way interaction term. As shown in Table S23, to compare these two models, the ANOVA results showed that reduced model achieved lower AIC and BIC values, indicating reduced model is a better-fitting model compared to the full model. And the p-value from the likelihood ratio test is greater than 0.05, indicating that the reduced model is not significantly different from the full model, suggesting that the reduced model is adequate. Therefore, although our results did not show a significant three-way interaction, we believe this still reflects, to some extent, the significant interaction between biotypes and medication.

**Table S23. Full model results of CPRS-psycho problem**

|  | Sum Sq | Mean Sq | F value | P value |
| --- | --- | --- | --- | --- |
| medcine | 0.25 | 0.25 | 0.60 | 0.44 |
| biotype | 3.4e-4 | 3.4e-4 | 8e-4 | 0.98 |
| week | 0.21 | 0.21 | 0.49 | 0.49 |
| med:biotp | 0.71 | 0.71 | 1.67 | 0.20 |
| med:week | 0.03 | 0.03 | 0.07 | 0.79 |
| week:biotype | 0.01 | 0.01 | 0.03 | 0.87 |
| week:biotype:med | 0.26 | 0.26 | 0.61 | 0.44 |

**Table S24. The ANOVA results of full model and reduced model**

|  | npar | AIC | BIC | logLik | deviance | Chisq | Df | Pr(>Chisq) |
| --- | --- | --- | --- | --- | --- | --- | --- | --- |
| model_reduced | 11 | 266.40 | 295.80 | -122.20 | 244.40 |  |  |  |
| model_full | 12 | 267.75 | 299.83 | -121.88 | 243.75 | 0.6407 | 1 | 0.4235 |

note: model_full: score ~ week * sub *med + (1 | ID)

model_reduce: score ~ week * sub + med * sub + week*med + (1 | ID)

**Reference**

1. Caseya, B.J., Cannoniera, T., Conleya, M.I., Cohenb, A.O. & Barchc, D.M. The Adolescent Brain Cognitive Development (ABCD) study: Imaging acquisition across 21 sites. *Developmental Cognitive Neuroence* **32**(2018).

2. Lees, B.*, et al.* Altered neurocognitive functional connectivity and activation patterns underlie psychopathology in preadolescence. *Biological Psychiatry: Cognitive Neuroscience and Neuroimaging* **6**, 387-398 (2021).

3. Lees, B.*, et al.* Association of prenatal alcohol exposure with psychological, behavioral, and neurodevelopmental outcomes in children from the adolescent brain cognitive development study. *American Journal of Psychiatry* **177**, 1060-1072 (2020).

4. Du, Y.*, et al.* NeuroMark: An automated and adaptive ICA based pipeline to identify reproducible fMRI markers of brain disorders. *NeuroImage: Clinical* **28**, 102375 (2020).

5. Bruna, J., Zaremba, W., Szlam, A. & Lecun, Y. Spectral Networks and Locally Connected Networks on Graphs. *Computer Science* (2013).

6. Kip F , T.N. & Welling, M. Semi-Supervised Classification with Graph Convolutional Networks. (2016).

7. Defferrard, M., Bresson, X. & Vandergheynst, P. Convolutional neural networks on graphs with fast localized spectral filtering. *Advances in neural information processing systems* **29**(2016).

8. Parisot, S.*, et al.* Disease prediction using graph convolutional networks: Application to Autism Spectrum Disorder and Alzheimer's disease. *Medical Image Analysis* **48**, 117-130 (2018).

9. Qiu, Y.*, et al.* Multi-channel Sparse Graph Transformer Network for Early Alzheimer’s Disease Identification. in *2021 IEEE 18th International Symposium on Biomedical Imaging (ISBI)* 1794-1797 (IEEE, 2021).

10. Wang, Y.*, et al.* MAGE: automatic diagnosis of autism spectrum disorders using multi-atlas graph convolutional networks and ensemble learning. *Neurocomputing* **469**, 346-353 (2022).

11. Vivar, G.*, et al.* Simultaneous imputation and classification using Multigraph Geometric Matrix Completion (MGMC): Application to neurodegenerative disease classification. *Artificial Intelligence in Medicine* **117**, 102097 (2021).

12. Fard, M.M., Thonet, T. & Gaussier, E. Deep k-Means: Jointly clustering with k-Means and learning representations. *Pattern Recognition Letters* (2020).

13. Gao, B., Yang, Y., Gouk, H. & Hospedales, T.M. Deep clustering with concrete k-means. (2019).
